# Attribution of Air Quality Benefits to Clean Winter Heating Policies in China: Combining Machine Learning with Causal Inference

**DOI:** 10.1021/acs.est.2c06800

**Published:** 2023-02-01

**Authors:** Congbo Song, Bowen Liu, Kai Cheng, Matthew A. Cole, Qili Dai, Robert J. R. Elliott, Zongbo Shi

**Affiliations:** # School of Geography, Earth and Environmental Science, University of Birmingham, Birmingham, B15 2TT, U.K.; ¶ Department of Economics, University of Birmingham, BirminghamB15 2TT, U.K.; § State Environmental Protection Key Laboratory of Urban Ambient Air Particulate Matter Pollution Prevention and Control, College of Environmental Science and Engineering, Nankai University, Tianjin300350, China; ∥ Department of Strategy and International Business, University of Birmingham, BirminghamB15 2TT, U.K.

**Keywords:** air pollution, winter heating, clean heating, causal inference, weather normalization, machine learning

## Abstract

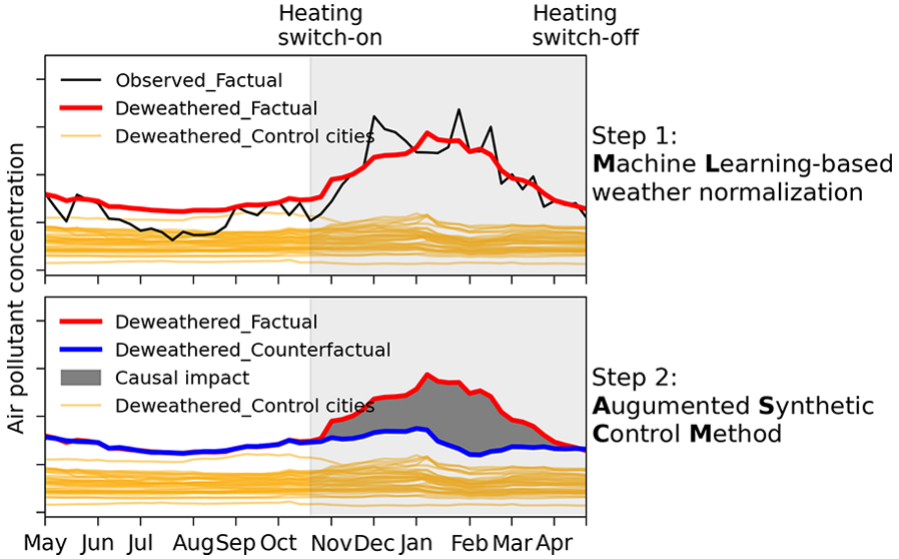

Heating is a major source of air pollution. To improve air quality, a range of clean heating policies were implemented in China over the past decade. Here, we evaluated the impacts of winter heating and clean heating policies on air quality in China using a novel, observation-based causal inference approach. During 2015–2021, winter heating causally increased annual PM_2.5_, daily maximum 8-h average O_3_, and SO_2_ by 4.6, 2.5, and 2.3 μg m^–3^, respectively. From 2015 to 2021, the impacts of winter heating on PM_2.5_ in Beijing and surrounding cities (i.e., “2 + 26” cities) decreased by 5.9 μg m^–3^ (41.3%), whereas that in other northern cities only decreased by 1.2 μg m^–3^ (12.9%). This demonstrates the effectiveness of stricter clean heating policies on PM_2.5_ in “2 + 26” cities. Overall, clean heating policies caused the annual PM_2.5_ in mainland China to reduce by 1.9 μg m^–3^ from 2015 to 2021, potentially avoiding 23,556 premature deaths in 2021.

## Abstract 2

Quasi-natural experimental study reveals significant benefits of clean heating policies on improving air quality and public health in China.

## 1 Introduction

**Figure 1:**
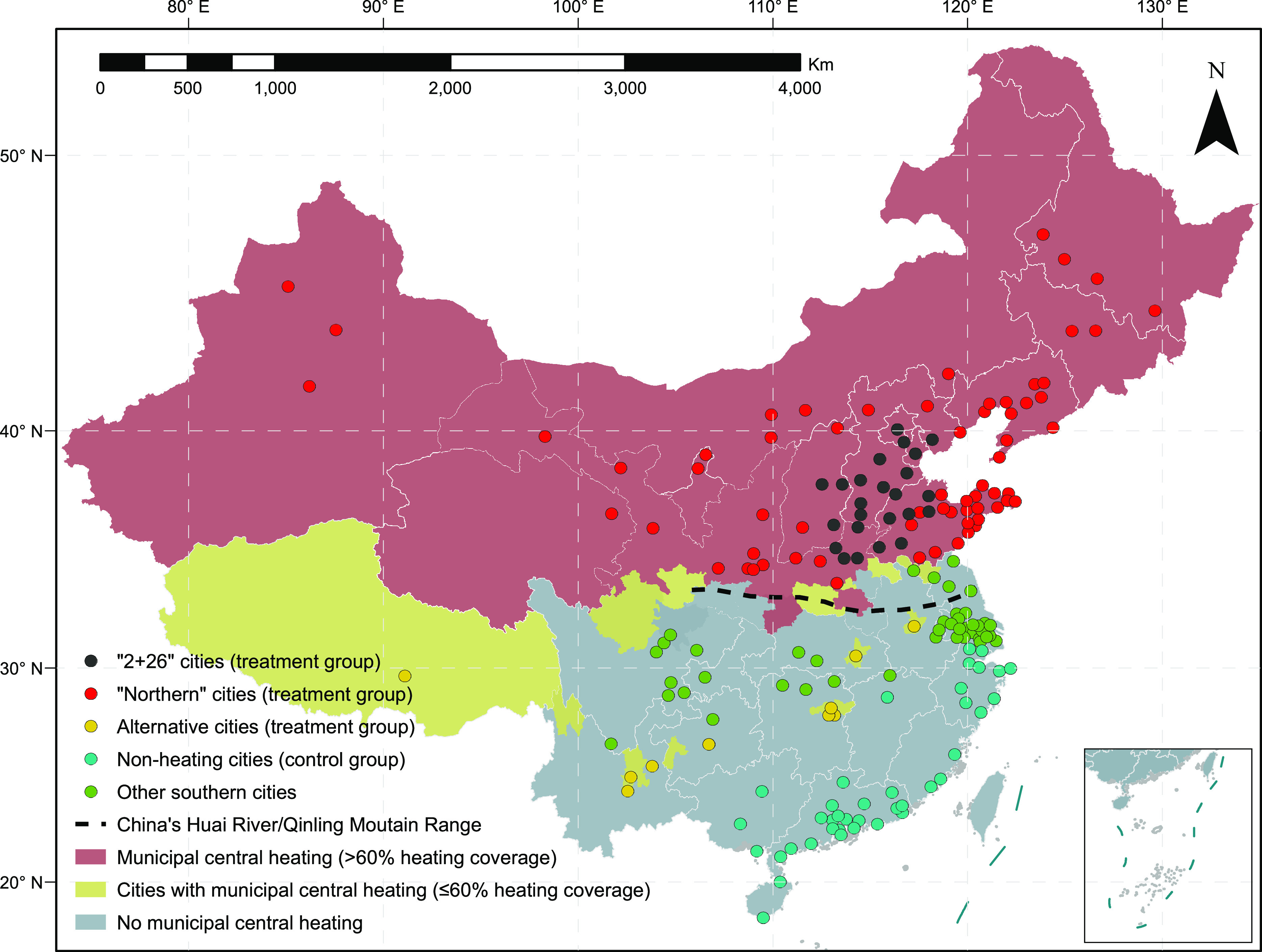
Geographic location of the studied cities. The 189 Chinese cities are categorized into five groups: (1) “2 + 26” cities (24 cities) with >60% heating coverage, (2) “northern” cities (66 cities) with >60% heating coverage, (3) alternative cities (9 cities)—southern cities with ≤60% heating coverage, (4) nonheating cities (39 cities)—southern cities with no central heating, and (5) the other southern cities (51 cities)—southern cities close to the heating cities. Note that the “northern” cities refer to the cities in northern China by excluding the “2 + 26” cities. Southern China/cities includes alternative cities, nonheating cities and the other southern cities. Mainland China includes all the 189 Chinese cities with data. The cities in each group are summarized in Table S1. Adapted from a base map with permission from National Basic Geographic Information Center, China (under open access license) (https://www.webmap.cn/commres.do?method=result100W).

China’s centralized winter heating strategy is one of the largest energy-consumption systems globally. In northern China (Figure 1), defined here as the north of the Huai River and Qinling Mountains, the policy provides free or heavily subsidized heating services to its urban residents during the predefined heating periods (known as the “Huai River Policy”). The central heating period is normally from November (heating switch-on) to March (heating switch-off). Coal is the main heating energy source in northern China, accounting for 83% of the total heating area in 2016.^1^ In addition to central heating, biomass burning was often used for heating in rural areas. Xiao et al.^2^ estimated that in 2014 heating from coal and biomass burning contributed 13% (5.6 μg m^–3^) and 43% (24.9 μg m^–3^) of the outdoor and indoor PM_2.5_ concentrations in urban China, respectively. It is found that coal and biomass burning are often associated with severe pollution episodes during the heating periods in northern China.^3,4^ Emission reductions from coal and biomass burning for winter heating lead to substantial benefits for both air quality and public health.^5−8^

In 2013, China introduced the “Air Pollution Prevention and Control Action Plan”,^9^ which accelerated the use of centralized/district heating and encouraged the switching to cleaner fuels including gas and electricity. In 2017, the Chinese central government issued the “Clean Winter Heating Plan for Northern China (2017–2021)”, which aimed to increase the share of clean heating in northern China to 50% by 2019 and to 70% by 2021 compared to the base scenario in 2016. In addition, the share of clean heating in “2 + 26 cities” (including Beijing, Tianjin, and 26 cities in Hebei, Shanxi, Shandong, and Henan provinces) should exceed 90% in urban areas, reaching 100% by 2021.^1^ In 2018, a new plan (3-year action plan to fight air pollution) was issued, and clean heating in northern China was among the major measures listed.^10^ All the above plans led to substantial emission reductions. Meanwhile, the annual average PM_2.5_ concentrations in Chinese cities within the national air quality monitoring network decreased from 72 μg m^–3^ in 2013 to 33 μg m^–3^ in 2020.^11^ Such improvement was mainly attributed to clean air actions including the promotion of clean fuels in the residential sector.^6^

Following the 5-year (2017–2021) implementation of clean heating in northern China, it is imperative to evaluate the effectiveness of the intensive heating-related policies. Chen et al.^12^ and Ebenstein et al.^13^ estimated that winter heating caused the total suspended particles (TSPs) and PM_10_ just north of the Huai River to increase by about 247.5 and 41.7 μg m^–3^, respectively, using Regression Discontinuity (RD) designs. However, the impacts over the cutoff of the discontinuity cannot represent locations far north of the Huai River. A difference-in-differences (DID) approach has also been applied to investigate the impacts of the clean winter heating plan.^14,15^ However, the DID model requires parallel trends between treatment and control groups during the pretreatment period. This means that the temporal trends of air pollutant concentrations in the control and treatment groups should share similar trends during nonheating periods. The complex weather effects and differences in emission trends often led to mismatch between two groups during nonheating periods. In addition, imprecise testing of the preparallel trend assumption can lead to biased estimation and misleading conclusions.^16^

A synthetic control approach, a recent improvement upon the DID, has been applied in social science studies and recognized as the most important innovation in the field of policy evaluation in recent years.^16,17^ The approach uses a weighted average of the set of control units to construct a more suitable comparison unit (i.e., synthetic unit) for the treatment unit. Thus, the difference in the trajectories between the synthetic and real treatment cities after the intervention started can be regarded as the causal impacts.

More recently, a new Augmented Synthetic Control Method (ASCM) was developed^18^ to improve the original synthetic control approach by removing the bias from any imbalance in pretreatment outcomes.

Our study estimates the causal impacts of winter heating on air quality in northern China using quasi-natural experimental designs. We follow a two-step approach (see Figure S1), coupling a machine learning-based weather normalization method with the ASCM model.^19^ First, we use a random forest-based machine learning model^20^ to decouple the effects of meteorology from observed air pollutant concentrations in 189 Chinese cities (Figure 1 and Table S1). Then the deweathered (normalized to multiyear average meteorological conditions) concentrations are fed into the ASCM model to isolate the causal impacts of winter heating on air quality. The improved approach provides the causal evidence on the impacts of air quality interventions/policies on annual average air pollutant concentrations in multiple cities under quasi-natural experimental designs, whereas previous studies focused on short-term interventions, such as the COVID-19 lockdowns.^19−21^

## 2 Materials and Methods

### 2.1 Quasi-natural Experimental Designs

The 189 Chinese cities are categorized into five groups (Figure 1 and Table S1): (1) “2 + 26” cities (24 cities) with >60% heating coverage, (2) “northern” cities (66 cities) with >60% heating coverage, (3) alternative cities (9 cities)—some southern cities with ≤60% heating coverage, (4) nonheating cities (39 cities)—southern cities with no central heating, and (5) other southern cities (51 cities)—southern cities close to the heating cities. Note that the “northern” cities are the cities in northern China having excluded the “2 + 26” cities. Population-weighted average air pollutant concentrations in “2 + 26” cities and “northern” cities are defined as two separate treatment groups whereas those in nonheating cities are regarded as the control units. The criteria of selecting control units include (1) no municipal central heating, (2) latitudes lower than the designated heating area in China, which is ∼31° N, and (3) less impacts from long-range transported pollution from the North China Plain.^22^ Alternative cities are similar to control cities in many aspects except that alternative cities are partially impacted by winter heating.

Two additional treatment groups are analyzed in this study: northern and mainland China. The northern China treatment group is constructed from population-weighted average air pollutant concentrations in all cities of “2 + 26” cities and “northern” cities whereas the mainland China treatment group is constructed from population-weighted average air pollutant concentrations in all monitoring cities (since 2014) in mainland China. To gain insights into city-level causal impacts of clean heating transition on air quality, 14 major Chinese cities (Beijing, Tianjin, Jinan, Shijiazhuang, Zhengzhou, Taiyuan, Xi’an, Lanzhou, Xining, Urumqi, Hohhot, Shenyang, Changchun, and Harbin) in northern China were also considered as treatment cities. The period and heating periods in each year are defined as pretreatment and post-treatment period separated by the start dates of winter heating (Table S2), respectively. The counterfactual air pollutant concentrations in all of the treatment groups are estimated from a weighted average of air pollutant concentrations in the cities of the control group using the ASCM. The heating impact is the difference in air pollutant concentrations between each treatment unit and its corresponding synthetic counterfactual unit.

### 2.2 Source of the Data

The city-level (national air quality report used) hourly air pollutant concentrations for ambient PM_2.5_, carbon monoxide (CO), sulfur dioxide (SO_2_) and nitrogen dioxide (NO_2_), ozone (O_3_) and 8-h average O_3_ (O_3__8h) from January 2014 to December 2021 for the 189 Chinese cities were retrieved from the open-access repository: https://quotsoft.net/air/. The hourly total gaseous oxidant (O_*x*_) is calculated by summing up the volume concentrations of NO_2_ and O_3_. The hourly measurements of ground-level meteorological conditions (including air temperature, relative humidity, wind speed, wind direction, and air pressure) at airports in the cities were obtained from the Integrated Surface Database (ISD) of NOAA (National Oceanic and Atmospheric Administration) using the “worldmet” R package. The hourly data on boundary layer height, total cloud cover, surface net solar radiation and total precipitation in each city were collected from ERA5 reanalysis data set (ERA5 hourly data on single levels from 1979 to present) from the European Centre for Medium-Range Weather Forecasts.

### 2.3 Weather Normalization

A random forest (RF)-based weather normalization approach was used to decouple the effects of meteorology from observed air pollutant concentrations in each city. The weather normalization was implemented using the “rmweather” R package developed by Grange and Carslaw.^23^ The weather normalized concentrations are also called “deweathered” concentrations in this study. The explanatory variables include meteorological variables and time variables. The meteorological variables include both ground-level measurements (i.e., air temperature, relative humidity, wind speed, wind direction, and air pressure) at airports in each city and ERA5 reanalysis data set (i.e., boundary layer height, total cloud cover, surface net solar radiation, and total precipitation). Time variables were added to the explanatory variables since they are proxies of time-related variables (e.g., emission intensity). Here, we used Unix time as a linear trend component, Gregorian date (day of the year) as a seasonal component, day of the week as a weekly component, and hour of the day as a diel-cycle component. In addition, we added “Lunar date” (day of the year according to Lunar calendar) as a time variable in the model to capture emission changes due to Chinese traditional holidays.^21^

The hyperparameters for the RF model are consistent with previous studies:^20,24,25^ a forest of 300 trees, n_tree = 300; and the minimum size of terminal nodes, min_node_size = 5. We use 70% of the original data set to train the model and the remaining 30% to test the model. The RF models for all the studied groups are with low bias (close to 0) and high correlation coefficients (>0.7), see Table S3. In addition, no obvious differences in the model performance for different groups are observed. For each air pollutant in each city, the deweathered concentration at a particular hour is calculated by averaging 1,000 predictions from the meteorological variables (excluding all time variables) randomly resampled from the observation period (2015–2021).^21^ The deweathered hourly concentrations are averaged into daily concentrations for all the air pollutants except O_3__8h. Daily maximum deweathered O_3__8h concentrations are calculated—deweathered MDA8 O_3_. The daily PM_2.5_, CO, SO_2_, NO_2_, O_3_, and MDA8 O_3_ are averaged into weekly data for ASCM analysis.

### 2.4 Augmented Synthetic Control Method

The purpose of the synthetic control method (SCM) is to construct an artificial control city (i.e., synthetic unit) that is comparable to the treatment city. The synthetic city is built from a weighted average of a group of control cities by reproducing air pollutant concentrations in the treatment city before the heating switch-on. The ASCM applied in our study extends from the SCM and uses an outcome model (ridge regression model) to estimate the bias due to imperfect preintervention fit and then directly corrects the original SCM estimate using the outcome model. Details regarding the ASCM are attached in the Supplementary Text S2. Results from ASCM show great improvements in the pretreatment match compared to that using uniform weights for cities in the control group (Table S4). The Ridge ASCM approach is implemented using the “augsynth” R package.^18^ A detailed description of the method can be found in Ben-Michael et al. (2021).^18^ The treatment groups and the control group are shown in the “Quasi-natural experimental designs” section. The Ridge ASCM is used on both observed and deweathered air pollutant concentrations on a year-by-year basis to isolate the effects from winter heating in each heating period. To allow for sufficient pretreatment periods in each year, the pretreatment period is from the end date (1 May) of the last heating period to the start date (range from October–November) of the current heating period in each city, and the post-treatment period is the current heating period (from Oct–Nov to 30 April next year).

## 3 Results

### 3.1 Air Pollution in Northern and Southern China

**Figure 2:**
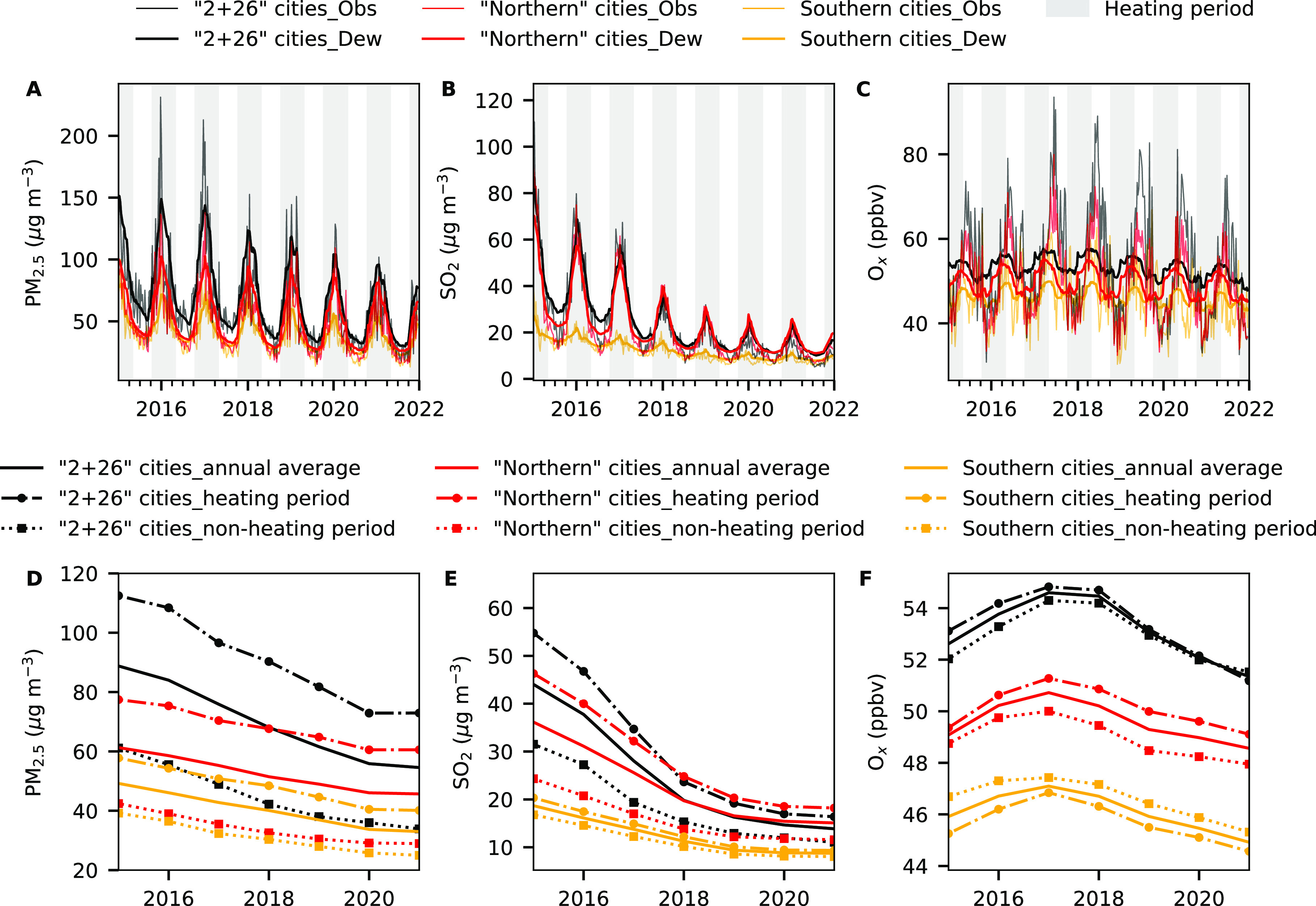
Air pollutant concentrations in “2 + 26” cities, “northern” cities, and southern cities. The subfigures in the top panel are observed and deweathered PM_2.5_, SO_2_, and O_*x*_ concentrations in “2 + 26” cities (in black), “northern” cities (in red), and southern cities (in yellow) at a weekly time resolution from January 2015 to December 2021. The shaded area in gray denotes the heating period. The subfigures in the bottom panel are interannual variations of deweathered air pollutant concentrations in “2 + 26” cities, “northern” cities, and southern cities during the heating period (dash-dot lines), the nonheating period (dotted lines), and the whole year (solid lines). Results for NO_2_, CO, and MDA8 O_3_ are shown in Figure S2.

As shown in Figures 2 and S2, higher levels of PM_2.5_, NO_2_, CO, O_3_, and O_*x*_ concentrations were observed in “2 + 26” cities than “northern” cities. In addition, significant reductions in PM_2.5_, SO_2_, NO_2_, and CO concentrations from 2015 to 2021 were observed across all regions. From 2015 to 2021, the annual mean observed PM_2.5_ concentration declined by 41.5 μg m^–3^ (from 85.6 to 44.1 μg m^–3^, by 48.6%) at a rate of −7.0 μg m^–3^ yr^–1^ (*R*^2^ = 0.98) in “2 + 26” cities, 21.2 μg m^–3^ (from 57.5 to 36.3 μg m^–3^, by 36.9%) at a rate of −3.4 μg m^–3^ yr^–1^ (*R*^2^ = 0.96) in “northern” cities, 19.3 μg m^–3^ (from 48.2 to 28.9 μg m^–3^, by 40.1%) at a rate of −3.3 μg m^–3^ yr^–1^ (*R*^2^ = 0.99) in southern cities and 24.2 μg m^–3^ (from 57.9 to 33.7 μg m^–3^, by 41.8%) at a rate of −4.1 μg m^–3^ yr^–1^ (*R*^2^ = 0.99) in mainland China. The decreasing rate of annual PM_2.5_ concentrations in “2 + 26” cities is ∼2.1 times that in “northern” cities. The annual SO_2_ concentrations in all regions decreased notably from 2015 to 2018 but less so after 2018 (Figure 2). A greater decline in observed SO_2_ concentrations was observed in “2 + 26” cities (from 42.7 to 17.9 μg m^–3^, −8.4 μg m^–3^ yr^–1^) than in “northern” cities (from 35.4 to 17.5 μg m^–3^, −5.9 μg m^–3^ yr^–1^) during 2015–2018. Consequently, SO_2_ concentrations in “2 + 26” cities after 2018 were lower than those in “northern” cities. The annual O_*x*_ concentrations in all the regions increased from 2014 to 2017/2018 and then decreased afterward.

From 2015 to 2021, the deweathered PM_2.5_ concentrations decreased by 34.2 μg m^–3^ (38.5%, from 88.8 to 54.6 μg m^–3^) for “2 + 26” cities, 15.6 μg m^–3^ (25.5%, from 61.3 to 45.7 μg m^–3^) for “northern” cities, 28.7 μg m^–3^ (36.7%, from 78.3 to 49.6 μg m^–3^) for northern China (including “2 + 26” cities and “northern” cities), and 19.6 μg m^–3^ (32.7%, from 60.0 to 40.4 μg m^–3^) for mainland China.

### 3.2 Air Pollution during Heating and Nonheating Periods

Clear seasonal cycles were found for both observed and deweathered air pollutant concentrations though observed values showed much greater fluctuation than weather-normalized ones (Figures 2 and S2). The start and end of the seasonal peaks in the deweathered concentrations coincided well with those of the heating periods (Figure 2, Table S2), agreeing with the enhanced emissions during heating periods. We estimated the weather effects on air pollutant concentrations by subtracting deweathered concentrations from observed concentrations. As shown in Figure S3, there is no obvious enhancement of weather effects on PM_2.5_, SO_2_, and CO concentrations during the heating periods compared to nonheating periods.

The deweathered PM_2.5_ concentrations during heating periods decreased by 39.6 μg m^–3^ (from 112.5 to 72.9 μg m^–3^, by 35.1%) at a rate of −7.3 μg m^–3^ yr^–1^ (*R*^2^ = 0.97) in “2 + 26” cities, 16.8 μg m^–3^ (from 77.4 to 60.6 μg m^–3^, by 21.8%) at a rate of −3.1 μg m^–3^ yr^–1^ (*R*^2^ = 0.97) in “northern” cities, 17.7 μg m^–3^ (from 57.8 to 40.1 μg m^–3^, by 30.6%) at a rate of −3.1 μg m^–3^ yr^–1^ (*R*^2^ = 0.98) in southern cities, and 21.8 μg m^–3^ (from 73.4 to 51.6 μg m^–3^, by 29.6%) at a rate of −3.9 μg m^–3^ yr^–1^ (*R*^2^ = 0.98) in mainland China. The declining rate of PM_2.5_ concentrations in “2 + 26” cities during heating periods is ∼2.4 times that in “northern” cities. However, such declines could not be entirely attributed to clean winter heating policies because substantial declines were also observed year by year during nonheating periods (Figure 2). The declining rates of deweathered PM_2.5_ concentrations during nonheating periods are −4.7 μg m^–3^ yr^–1^ (*R*^2^ = 0.96) in “2 + 26” cities, −2.3 μg m^–3^ yr^–1^ (*R*^2^ = 0.94) in “northern” cities, −2.4 μg m^–3^ yr^–1^ (*R*^2^ = 0.97) in southern cities, and −2.9 μg m^–3^ yr^–1^ (*R*^2^ = 0.96) in mainland China. However, the declining rates of deweathered PM_2.5_ concentrations in “2 + 26” cities, “northern” cities, southern cities, and mainland China during heating periods are −2.6, −0.8, −0.7, and −1.0 μg m^–3^ yr^–1^ greater than those during nonheating periods, respectively. Such effects are more obvious for SO_2_ and CO (Figure S2). The difference between deweathered O_*x*_ during heating and nonheating periods became increasingly smaller for “2 + 26” cities (from 1.1 to −0.3 ppbv) but increasingly larger for “northern” cities (from 0.6 to 1.2 ppbv) from 2015 to 2021, implying a larger decline in atmospheric oxidants during heating period in “2 + 26” cities than in “northern” cities. The interannual variations of air pollutant concentrations in “2 + 26” cities were different to those in “northern” cities. Therefore, “2 + 26” cities and “northern” cities should be studied separately regarding the impacts of winter heating on air quality.

### 3.3 Region-Level Synthetic Differences

**Figure 3:**
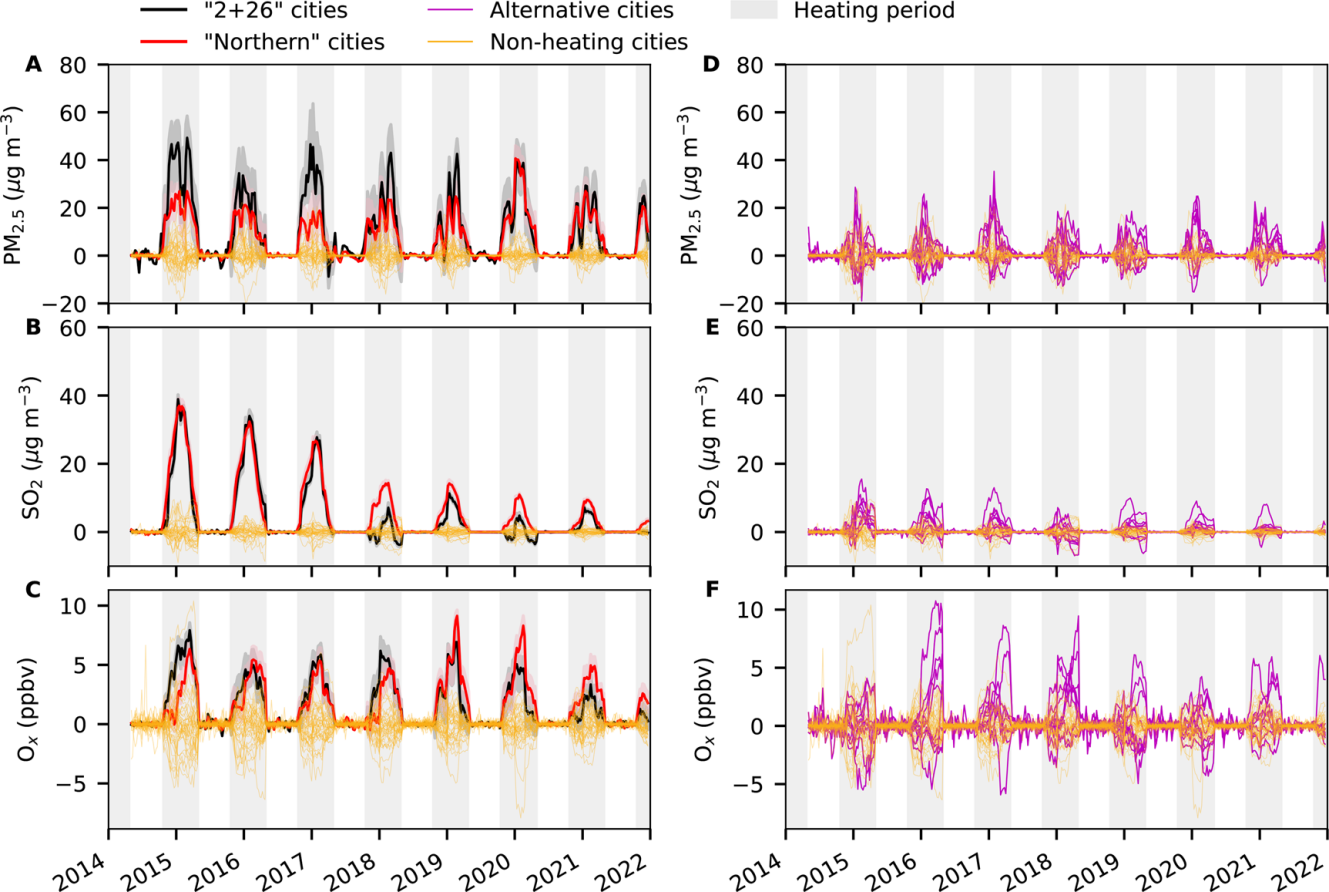
Causal impacts of winter heating on deweathered PM_2.5_, SO_2_, and O_*x*_ for the treatment groups and in-place placebo tests for cities in the control group and alternative cities. The results for “2 + 26” cities and “northern” cities are shown in the left panel whereas those for alternative cities are shown in the right panel. The in-place placebo tests for each city in the control group are shown in yellow. The shaded area of the effect from Ridge ASCM for “2 + 26” cities and “northern” cities denotes the pointwise 95% confidence interval using Jackknife+ procedure. The shaded area in gray denotes the heating period. Results for all the studied air pollutants (i.e., PM_2.5_, SO_2_, NO_2_, CO, MDA8 O_3_, and O_*x*_) and all the treatment groups (“2 + 26” cities, “northern” cities, northern China, and China) are shown in Figure S7.

The “2 + 26” cities and “northern” cities were regarded as two “treatment” groups (i.e., with winter heating) and the 39 nonheating cities in southern China were treated as a control group (i.e., without winter heating) (Figure 1 and Table S1). The synthetic differences (i.e., the difference between the factual and counterfactual concentrations, Figure S4) in deweathered air pollutant concentrations during nonheating periods are close to 0 (Figures 3 and S5 and Table S4), suggesting that the synthetic control group can closely reproduce the deweathered trend (i.e., successfully minimized the difference) of the treatment group during nonheating periods. This is what we expect because there is no heating during nonheating periods in the treatment groups. However, we observed large differences in the synthetic difference of observed concentrations for all air pollutants during nonheating periods (Figure S6 and Table S5).

**Figure 4:**
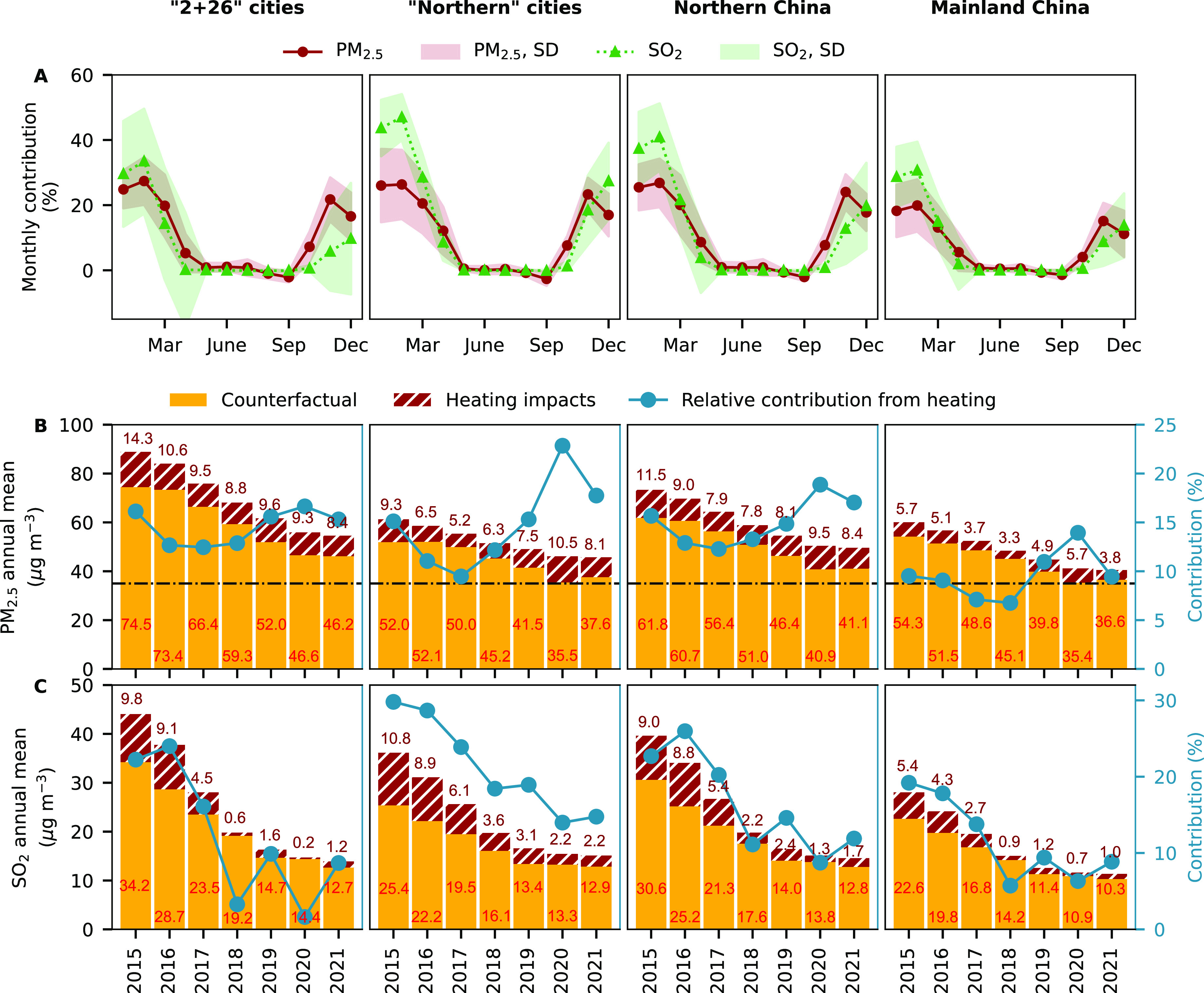
Temporal variations of contributions from winter heating to PM_2.5_ and SO_2_ concentrations. The panels from left to right show results from “2 + 26” cities, “northern” cities, northern and mainland China, respectively. The subfigures in the (A) top panel are results for monthly variations of average contributions from winter heating to deweathered PM_2.5_ and SO_2_. The shaded area in the subfigures of the top panel denote the standard deviation (SD) of the data points. The subfigures in the middle and the bottom panel are results from contributions from winter heating to annual average deweathered (B) PM_2.5_ and (C) SO_2_, respectively. The counterfactual annual average deweathered concentrations and the contribution of winter heating to annual average deweathered concentrations in each year are noted on each subfigure. The relative contributions from winter heating to annual average deweathered concentrations are shown in the right axis. The horizontal dash-dotted line in black in the middle panel denotes the World Health Organization interim target-I (35 μg m^–3^). All subfigures in the top/middle/bottom panel share same left-axis/right-axis. Results for the other pollutants and other regions are shown in Table S6.

Overall, the synthetic difference in deweathered concentrations during heating periods for PM_2.5_ and SO_2_ peaked in January and February regardless of the regions (Figure 4). During heating periods, winter heating contributed as high as 37.4 ± 11.3% and 28.7 ± 9.2% to monthly average SO_2_ in northern and mainland China during 2015–2021, respectively. The highest contributions to monthly average PM_2.5_—26.8 ± 7.4% in northern China and 19.9 ± 8.0% in mainland China—were observed in February. On average, the annual synthetic difference in deweathered PM_2.5_ concentrations during 2015–2021 was 10.1 ± 2.0 μg m^–3^ (decreased from 14.3 μg m^–3^ in 2015 to 8.4 μg m^–3^ in 2021) in “2 + 26” cities, and 7.6 ± 1.8 μg m^–3^ (decreased from 9.3 μg m^–3^ in 2015 to 8.1 μg m^–3^ in 2021) in “northern” cities (Figure 4 and Table S6). During 2015–2021, such annual synthetic difference in deweathered SO_2_ was 3.9 ± 4.0 μg m^–3^ in “2 + 26” cities and 5.3 ± 3.4 μg m^–3^ in “northern” cities, respectively. Note that the annual synthetic difference in deweathered SO_2_ declined substantially from 2015 to 2018—from 9.8 to 0.6 μg m^–3^ in “2 + 26” cities and from 10.8 to 3.6 μg m^–3^ in “northern cities”—but showed a small change from 2018 to 2021. The annual synthetic difference in deweathered CO (Figure S7 and Table S6) was 0.17 ± 0.06 mg m^–3^ in “2 + 26” cities and 0.12 ± 0.02 mg m^–3^ in “northern” cities, respectively. The annual synthetic difference in deweathered NO_2_ concentration (Figure S7 and Table S6) was close to zero in both “2 + 26” cities (0.0 ± 1.0 μg m^–3^) and “northern” cities (−0.3 ± 0.2 μg m^–3^). And those in deweathered MDA8 O_3_ and O_*x*_ (Figure S7 and Table S6) were 5.3 ± 1.9 μg m^–3^ and 1.5 ± 0.5 ppbv in “2 + 26” cities, 4.4 ± 0.6 μg m^–3^ and 1.6 ± 0.3 ppbv in “northern” cities, respectively.

### 3.4 City-Level Synthetic Differences

**Figure 5:**
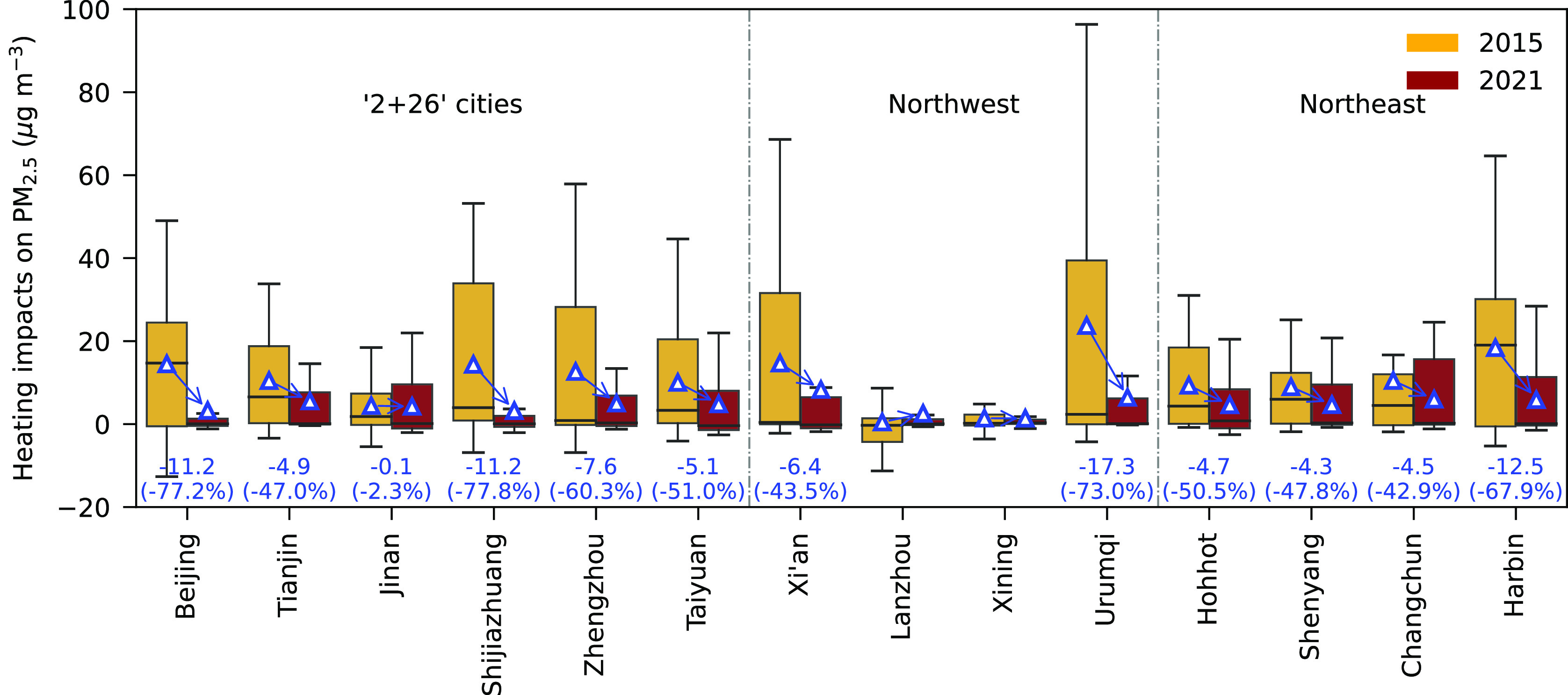
Box plots of heating impacts on PM_2.5_ in 14 major cities in northern China in 2015 versus 2021. Box plots are based on weekly data points, including both heating and nonheating periods. Lower and upper box boundaries represent 25th and 75th percentiles, respectively; line and triangle inside box represent median and mean, respectively; lower and upper error lines represent 1.5*IQR (interquartile range) below the third quartile and above the first quartile, respectively. Changes and percentage changes in annual PM_2.5_ concentrations in each city between 2015 and 2021 are noted on the figure. No obvious changes for Lanzhou and Xining from 2015 to 2021, due to already low heating impacts since 2015. Time series of the synthetic difference in deweathered weekly PM_2.5_ concentrations in the 14 major northern cities are shown in Figure S8. Heating impacts on annual PM_2.5_, SO_2_, and CO in the 14 major cities in northern China from 2015 to 2021 are summarized in Table S7.

During 2015–2021, the larger annual synthetic difference in deweathered PM_2.5_ (Figure 5, Figure S8, and Table S7) was found in Urumqi (18.8 ± 6.1 μg m^–3^), followed by Xi’an (16.2 ± 4.4 μg m^–3^), Harbin (13.3 ± 5.7 μg m^–3^), Shijiazhuang (12.0 ± 6.3 μg m^–3^), Changchun (10.5 ± 2.9 μg m^–3^), and Zhengzhou (10.3 ± 3.5 μg m^–3^). Among the 14 cities, the three cities with lowest annual synthetic differences in deweathered PM_2.5_ (Table S7) were Lanzhou (0.7 ± 3.0 μg m^–3^), Xining (2.3 ± 1.6 μg m^–3^), and Jinan (6.0 ± 1.8 μg m^–3^). Except for Lanzhou, Xining, and Jinan, the other cities all showed markable decreases, with percentage decrease greater than −40%, in annual synthetic differences in deweathered PM_2.5_ (Figure 5). The largest percentage decrease in annual synthetic difference in deweathered PM_2.5_ from 2015 to 2021 (Figure 5) was observed in Shijiazhuang (−77.8%, from 14.4 to 3.2 μg m^–3^), followed by Beijing (−77.2%, from 14.5 to 3.3 μg m^–3^), Urumqi (−73.0%, from 23.7 to 6.4 μg m^–3^), Harbin (−67.9%, from 18.4 to 5.9 μg m^–3^), and Zhengzhou (−60.3%, from 12.6 to 5.0 μg m^–3^). In 2021, annual synthetic differences in deweathered PM_2.5_ were still as high as 8.3 μg m^–3^ in Xi’an, 6.4 μg m^–3^ in Urumqi, 6.0 μg m^–3^ in Changchun, and 5.9 μg m^–3^ in Harbin.

The five cities with the largest annual synthetic differences in deweathered SO_2_ during 2015–2021 (Table S7) were Taiyuan (14.8 ± 11.4 μg m^–3^), Harbin (7.7 ± 4.0 μg m^–3^), Shenyang (7.3 ± 7.7 μg m^–3^), Hohhot (7.1 ± 4.2 μg m^–3^), and Changchun (6.4 ± 4.3 μg m^–3^). The five cities with largest percentage decreases in annual synthetic difference in deweathered SO_2_ from 2015 to 2021 were observed in Zhengzhou, Shijiazhuang, Tianjin, Shenyang, and Beijing (Table S7), with percentage decrease greater than 93%. In 2021, annual synthetic differences in deweathered SO_2_ in all the 14 cities decreased to less than 3.5 μg m^–3^, with highest values in Taiyuan (3.5 μg m^–3^), Hohhot (2.0 μg m^–3^), and Lanzhou (1.8 μg m^–3^).

## 4 Discussion

### 4.1 Causal Inference of “Interventions”

To estimate the causal impacts of a policy “intervention” or “treatment”, such as those of winter heating on air quality in northern China, the key is to construct counterfactual (business as usual) trajectories. Traditionally, in air quality intervention studies, the counterfactual concentrations were simulated by chemical transport models (CTMs). However, CTMs rely heavily on timely and accurate emission inventory,^26^ which are often not available especially in scenarios with abrupt emission changes, such as COVID lockdowns^20^ and extensive clean winter heating policies (this study). Recently, Shi and Song et al.^20^ proposed a combined deweathering and “detrending” approach to disentangle the changes due to both meteorological and “counterfactual” variations (confounding factors) from the intervention-associated changes in emissions to understand the resulting differences in air pollutant concentrations. In that study, deweathered changes before and after the intervention date in previous years served as “counterfactual” deweathered changes. However, such an approach is not suitable for evaluating the impacts of winter heating on air quality since the counterfactual air pollutant concentrations varied day by day during heating periods and for each year. This is because emissions are constantly changing, making it impossible to “detrend”.

DID is a popular model to evaluate interventions. However, by examining the observed and deweathered differences in air pollutant concentrations between heating and nonheating cities, the paired groups (i.e., the treatment groups and the control groups) during pretreatment periods (i.e., nonheating periods for each year) did not meet the parallel trend assumption for the DID model, especially for “2 + 26” cities (Figure S6). This is because the emission intensity and meteorology are different for the paired groups during nonheating periods. Thus, the widely used DID model may lead to biased estimation. RD designs have also been utilized to study air quality impacts from winter heating switch-on/off^27,28^ and China’s Huai River Policy on air pollution.^12,13^ However, changes in air quality over the cutoff points cannot represent changes of the whole heating period or the full year or the overall impacts of winter heating on air quality. The intensity of the heating, and thus emissions, changes on a daily basis. Furthermore, even the deweathered differences in the RD design cannot be attributed to winter heating due to different average meteorological conditions between heating and nonheating cities, partially resulting in the mismatch (i.e., ≠ ∼0) of air quality trends between the paired groups during nonheating periods (Figure S6).

The synthetic control approach can minimize the bias from differences in concentrations between treatment (e.g., northern) and control (e.g., southern) cities during nonheating periods (Table S4). But this cannot be based on observed concentrations because such synthetic difference is highly variable due to meteorological variability (Figure S5) and the resulting high mismatch in concentrations during nonheating periods (Table S5). This is confirmed by the large differences in the synthetic difference of observed concentrations during nonheating periods between paired groups, leading to large bias in the estimated effects during heating periods (Figure S5 and Table S5). As we can see from Table S6, conclusions made from ASCM based on observed concentrations could be completely different to those based on deweathered ones, and thus, they could be highly misleading for air quality management. We conclude that it is essential to remove the meteorology-induced variations in both the treatment and the control group before applying synthetic control techniques.^29^

The synthetic differences in deweathered SO_2_, PM_2.5_, SO_2_, CO, MDA8 O_3_ and O_*x*_ concentrations during heating periods were substantial, but there remains the possibility that such observable synthetic differences were caused by differences between the treatment and control groups that we may not know, in addition to those due to the winter heating. For this reason, we conducted a series of placebo tests to validate the robustness of our estimates.^30^ First, we assume that each city in the control group (southern cities) has winter heating just like the treatment (“2 + 26”/“northern”) cities. We applied the same approach to the 39 control cities one by one. The synthetic differences in each nonheating city and the control cities are shown in Figures 3 and S7. We observed a clear distinction between the synthetic difference in PM_2.5_, SO_2_, CO, MDA8 O_3_, and O_*x*_ for the nonheating cities and that for “2 + 26” cities and “northern” cities from in-place placebo tests (Figure S7), demonstrating that our results for “2 + 26” and “northern” cities are not driven by chance and can be attributed to the winter heating-related emissions. In addition, the synthetic differences in NO_2_ concentrations in “2 + 26” cities and “northern” cities were similar to those in the nonheating cities (Figure S7), indicating that winter heating in northern China has less of an impact on NO_2_ than the other air pollutants, as expected.

To further validate that the synthetic difference is attributable to the winter heating impacts, we applied the same approach to selected southern cities with ≤60% municipal heating coverage as alternative treatment units. Most of the alternative cities showed larger enhancements in PM_2.5_, SO_2_, and O_*x*_ during heating periods than for the nonheating cities (Figure 3), suggesting that the enhanced synthetic differences during heating periods in “2 + 26” cities and “northern” cities can be attributed to the winter heating-related emissions.

In conclusion, our quasi-natural experimental designs with the improved approach by combining machine learning (ML) and ASCM (ML-ASCM), can reliably attribute the causal effects to “interventions”, such as the winter heating and clean heating policies on air quality (Table S6). The limitations of using a ML-ASCM are discussed in Supplementary Text S2.

### 4.2 Impacts of Heating Policies on Air Quality

Overall, winter heating during 2015–2021 causally increased annual PM_2.5_ by 8.9 ± 1.3 μg m^–3^, SO_2_ by 4.4 ± 3.3 μg m^–3^, CO by 0.14 ± 0.05 mg m^–3^, MDA8 O_3_ by 5.1 ± 1.5 μg m^–3^, and O_*x*_ by 1.6 ± 0.3 ppbv (equal to synthetic differences) in northern China. No obvious effects (−0.2 ± 0.5 μg m^–3^) of winter heating on NO_2_ were observed in northern China. Averaging over mainland China, winter heating contributed to annual PM_2.5_ by 4.6 ± 1.0 μg m^–3^, SO_2_ by 2.3 ± 1.9 μg m^–3^, CO by 0.07 ± 0.02 mg m^–3^, MDA8 O_3_ by 2.6 ± 0.4 μg m^–3^, and O_*x*_ by 0.9 ± 0.2 ppbv. Winter heating contributed 15.0 ± 2.2% and 9.5 ± 2.2% to the annual PM_2.5_ concentrations in northern China and mainland China, respectively. Our estimated contribution from heating to ambient PM_2.5_ in mainland China in 2015 was 5.7 μg m^–3^. This is almost the same as that estimated by Yun et al.^2^ based on emission inventory and CTM (5.6 μg m^–3^) for 2015.^24,31^ However, our approach is orders of magnitude more computationally efficient than CTM. Furthermore, uncertainty concerning the ML-ASCM approach is well quantified. Our data-driven approach may underestimate the contribution of winter heating to air pollution as there are short-term air quality interventions (e.g., red alerts) in winter in certain areas of northern China compared to those in southern China. However, such impacts should contribute little to annual synthetic differences compared to winter heating, as shown from small annual synthetic differences in deweathered NO_2_ concentrations (Figure S7 and Table S6). If other sources or interventions have substantial impacts on our analysis, we should see non-zero synthetic differences in NO_2_.

The winter heating driven PM_2.5_ and SO_2_ concentrations peaked around January/February, which coincided with the normal peak of the Heating Degree Days index (HDD, an indication of the energy consumption demand for heating), further confirming that the enhancement of heating emissions was causally driven by increased heating supply. Our study shows that the contribution from winter heating to annual average PM_2.5_ in mainland China decreased from 5.7 μg m^–3^ in 2015 to 3.8 μg m^–3^ in 2021, demonstrating the effectiveness of the clean winter heating policies. In particular, the heating impacts on PM_2.5_ in “2 + 26” cities during 2015–2021 decreased by 5.9 μg m^–3^ (41.3%). The decreasing rate (−2.6 μg m^–3^ yr^–1^ from 2015 to 2017) is higher than that estimated by Zhang et al.^6^ (−1.8 μg m^–3^ yr^–1^) from CTM, noting that their estimates are from 2013 to 2017.

Our study reveals that the contributions of winter heating to air pollution in “2 + 26” cities and “northern” cities have different trends from 2015 to 2021. Overall, the effects of winter heating on PM_2.5_ in “2 + 26” cities are 2.4 ± 2.3 μg m^–3^ higher than those in “northern” cities, though it accounted for comparable fractional contributions to PM_2.5_ (14.5 ± 1.7% vs 14.8 ± 4.2%). The benefit of clean heating on declining PM_2.5_ during 2015–2021 in “2 + 26” cities, 5.9 μg m^–3^ (41.3%), is much larger than the 1.2 μg m^–3^ (12.9%) in “northern” cities (Figure 4). The heating impacts on SO_2_ in “2 + 26” cities were 1.4 ± 1.0 μg m^–3^ lower than those in “northern” cities. This is mainly because lower sulfur content in coal in “2 + 26” cities than “northern” cities. The coal with high sulfur content (>1%) was rarely used in “2 + 26” cities, but it was still used in surrounding areas, especially in rural areas. Now, the sulfur content in bituminous coal is basically lower than 0.5%. The local standard is even lower, and the requirements for sulfur content in briquette coal in Tianjin are <0.4%. Stricter policies in “2 + 26” cities (than “northern” cities) led to lower winter heating emissions and thus causing a reduced impact of heating emissions on SO_2_. These results demonstrate clear air quality benefits from the stricter clean heating policies in “2 + 26” cities.^1^ The lowest heating impacts on PM_2.5_ during the study period were found in 2018 for “2 + 26” cities and in 2017 for “northern” cities (Figure 4) due to the strict “coal to gas” policy, which partially resulted in the natural gas shortages in northern China.^31^ The highest relative contributions of heating to annual PM_2.5_ in both northern China (18.9%) and mainland China (12.9%) were found in 2020. One of the possible reasons is the increased usage of household fuel for heating during the national COVID-19 quarantine in 2020.^32,33^ On the other hand, the relative contribution of winter heating to PM_2.5_ increased from 2017 to 2020 in “2 + 26” and “northern” cities, reflecting a rebound in heating-related emissions after the gas shortage during 2017–2018, as well as the effective emission reductions from other sectors, as shown in the changes in counterfactual concentrations (Figure 4).

We show that winter heating has significantly contributed to air quality and health inequalities in northern vs southern China (Figures 3 and S7). However, the clean heating policies have reduced such inequalities. In 2015, the average concentration of PM_2.5_ in northern China is ∼40.2% higher than that in the southern China. In 2021, this difference has reduced to ∼27.2%, partially due to the clean winter heating policies.

### 4.3 Implications

Of all the air pollution control measures,^6^ the clean winter heating policies accounted for ∼10.8% (from 11.5 to 8.4 μg m^–3^) and ∼9.7% (from 5.7 to 3.8 μg m^–3^) of the decline in annual PM_2.5_ in northern (from 78.3 to 49.6 μg m^–3^) and mainland China (from 60.0 to 40.4 μg m^–3^) during 2015–2021 (Figure 4). Despite the decreasing PM_2.5_ concentrations year by year in China, the relative contribution from winter heating to PM_2.5_ (Figure 4) and the attributable fraction for PM_2.5_ exposure-associated diseases (Figure S9) show little decrease from 2015 to 2017/2018 (natural gas shortages) and rebounded after 2017/2018 (“coal to gas” was urgently stopped) and then reached the maximum in 2020 but dropped thereafter. Overall, the attributable fraction for premature deaths dropped from ∼1.7% in 2015 to ∼1.5% in 2021 (Supplementary Text S3), corresponding to ∼169,016 and ∼145,460 premature deaths, respectively, indicating that clean heating policies potentially avoided 23,556 premature deaths in 2021 (using 2015 as the baseline).

The heating impacts on SO_2_ and CO showed little changes after 2018, reaching 1.7 μg m^–3^ and 0.08 mg m^–3^ in 2021 in northern China. There is still potential to further reduce primary emissions from heating in northern China (particularly for Xi’an, Urumqi, Changchun, Harbin, Figures 4 and 5), but such scope is limited for “2 + 26” cities (Figure 4). Furthermore, the reduction rate of the heating impact on PM_2.5_ (−27.0%) in northern China from 2015 to 2021 is much smaller than that for SO_2_ (−81.1%) (Figure 4). Such a disproportional decrease is likely due to the dominant contribution of secondary aerosol to PM_2.5_, which responds nonlinearly to changes in primary precursors such as SO_2_.^34,35^ The synthetic difference in O_*x*_ has a positive impact on that in PM_2.5_ for both “2 + 26” cities and “northern” cities, whether based on observed or deweathered concentrations, regardless of using a single-pollutant or multipollutant regression (Table S8). The enhanced impacts from heating on O_*x*_ during heating periods were mainly caused by heating-related net-production of O_3_ since synthetic difference in O_3_ has increased and that in NO_2_ has been stable (Figure S7). Such effects might be related to increased volatile organic compound (VOC) emissions from natural gas and/or coal combustion during the heating periods^36^ and/or nonlinear photochemical processes in the atmosphere.^26^ In addition, unburned natural gas in gas distribution systems and residences also contribute to emissions of aromatic VOC species with high hydroxyl radical (^•^OH) reactivities.^37^ Increase in O_3_ contributes to higher atmospheric oxidation capacity, facilitating the formation of secondary PM_2.5_.^43^ This is consistent with a recent study demonstrating that strict VOC control in winter is critical to mitigate PM_2.5_ more efficiently by reducing O_*x*_ simultaneously.^38^ Future modeling studies are required to further investigate the impacts of heating emissions on O_3_ as well as O_*x*_ during heating periods in northern China.

Our results show low heating impacts on air pollution in Lanzhou, Xining, and Jinan after 2015 (Figure 5). This is mainly because the three cities implemented the elimination and nature gas transformation of coal fired boilers during 2012–2014, ahead of the other northern cities (Figure S8). In addition, Jinan accelerated the development of geothermal resources, in addition to nature gas and electricity, to solve the problem of urban heating owing to its abundant geothermal resources. The large declines in heating impacts on annual PM_2.5_ in all the other northern cities from 2015 to 2021 further supported the substantial environmental benefits of clean heating policies. Switching to greener energy sources, such as heat pumps and geothermal resources, would be important for reducing heating-related secondary pollution (i.e., PM_2.5_ and O_3_) and contributing to carbon neutrality.^39^

Winter heating during 2015–2021 accounted for ∼2.8% and ∼1.6% of premature deaths in northern and mainland China (Supplementary Text S3), respectively. In addition, winter heating may also contribute to excess mortality associated with O_3_ exposure. Thus, abating heating-related secondary pollution has further potential health benefits in northern China. Note that the population-weighted annual average PM_2.5_ (observed: 39.7 μg m^–3^, deweathered: 49.6 μg m^–3^) in northern China in 2021 was still far above 35 μg m^–3^, the World Health Organization interim target-I (WHO IT-1). In the counterfactual scenario in 2021, population-weighted annual deweathered PM_2.5_ concentrations in “2 + 26” cities, as well as northern China did not meet the WHO IT-1 (Figure 4), implying that other emission sources in addition to winter heating need to be tackled. Note that winter heating-related annual average PM_2.5_ concentration in northern China, alone, during 2015–2021 (PM_2.5_: 7.8–11.5 μg m^–3^) are always far above the new annual Air Quality Guideline level (PM_2.5_: 5 μg m^–3^) recommended by WHO,^40^ implying that the potential to abate heating related PM_2.5_ and thus improve public health remains significant,^41,42^ but the effectiveness of future heating policies likely depends on controlling the emissions of gaseous precursors.

## Data Availability

Air quality data can be retrieved from the open-access repository: https://quotsoft.net/air/. Data and codes for the augmented synthetic control method can be retrieved from the open-access repository: https://github.com/songnku/ML-ASCM. Data and codes used in this study are also available upon request from the corresponding authors.

## Supporting Information Available

Augmented synthetic control method (Text S1); limitation of the ML-ASCM (Text S2); PM_2.5_-related health effects from winter heating (Text S3); concept for estimating the causal impacts (Figure S1); machine-learning based weather normalization (Figure S2–S3, S6, and Table S3); synthetic control method (Figure S4, S5, S7, S8 and Tables S4–S7); attributable fractions for different diseases (Figure S9); selection of the cities (Table S1–S2); and OLS regressions between synthetic difference in PM_2.5_ against other pollutants (Table S8) (PDF)
