# Supplementary material for: Attribution of Air Quality Benefits to Clean Winter Heating Policies in China: Combining Machine Learning with Causal Inference
Source: Environ Sci Technol. 2023 Feb 1;57(46):17707–17. doi: 10.1021/acs.est.2c06800 (PMC10666544; doi:10.1021/acs.est.2c06800)
Supplement: Supplementary file 1 — es2c06800_si_001.pdf [file es2c06800_si_001.pdf]

Supporting Information for

Attribution of air quality benefits to clean winter heating polices in China: Combining machine learning and causal inference

Congbo Song[a,e], Bowen Liu[b,d], Kai Cheng[b,f], Matthew A. Cole[b], Qili Dai[c,*], Robert J. R. Elliott[b] and Zongbo Shi[a,*]

[a]School of Geography, Earth and Environmental Science, University of Birmingham, Birmingham, B15 2TT, UK

[b]Department of Economics, University of Birmingham, Birmingham B15 2TT, UK

[c]State Environmental Protection Key Laboratory of Urban Ambient Air Particulate Matter Pollution Prevention and Control, College of Environmental Science and Engineering, Nankai University, Tianjin 300350, China

[d]Department of Strategy and International Business, University of Birmingham, Birmingham B15 2TT, UK

[e]Present address: National Centre for Atmospheric Science (NCAS), University of Manchester, Manchester M13 9PL, UK

[f]Present address: Institute of Economics, School of Social Sciences, Tsinghua University, Beijing 100084, China

*Email: Z.Shi@bham.ac.uk (Z.S.), daiql@nankai.edu.cn (Q.D.)

This PDF file (23 pages) includes:

Supplementary Text S1 to S3
Text S1 to S3
Figures. S1 to S9
Tables S1 to S8
SI References

Supporting Information Text

Text S1. Augmented synthetic control method.

Suppose the post-treatment air pollutant concentrations if unexposed to the intervention at period T can be modelled as below:

$Y_{iT} = \eta_0 + \boldsymbol{X}'_i\boldsymbol{\eta} + \varepsilon_{iT}$ [1]

where i denotes unexposed unit, $\boldsymbol{X}_i$ is $(T_0 \times 1)$ vector containing lagged air pollutant concentrations for pre-treatment periods in which T_0 is the number of pre-treatment weeks, ε is the noise term. According to the Ridge regression, we can obtain Ridge estimators for parameters $\hat{\eta}_0^{ridge}$ and $\hat{\boldsymbol{\eta}}^{ridge}$. Then the Ridge ASCM estimator is

$\hat{Y}_{1T} = \sum \hat{w}_j^{scm} Y_{jT} + \left(\boldsymbol{X}_1 - \sum \hat{w}_j^{scm}\boldsymbol{X}_j\right) \cdot \hat{\boldsymbol{\eta}}^{ridge} = \sum \hat{w}_j^{aug} Y_{jT}$ [2]

where 1 denotes the notation for treatment city and j denotes the notation for control cities. $\hat{w}_j^{aug}$ represents the estimated element of the targeted weights vector $\boldsymbol{W}^{aug}$. In addition, $\hat{w}_j^{scm}$ represents the estimated weight from the original SCM. The details for the weight vector are shown in the following section. In this regard, $\hat{Y}_{1T}$ can be considered as the synthetic counterfactual air pollutant concentrations for treatment city after the heating switch-on. Then, the causal impacts (ΔY) of winter heating on air quality can be estimated through the difference between the factual air pollutant concentrations (Y_{1T}) and synthetic counterfactual air pollutant concentrations ($\hat{Y}_{1T}$) as below:

$\Delta Y = Y_{1T} - \hat{Y}_{1T}$ [3]

Annual mean of ΔY, including non-heating and heating periods, can thus indicate the contribution of winter heating to annual mean of air pollutant concentrations.

The synthetic control method (SCM) was proposed to estimate the effects of policy interventions that are implemented at aggregate levels like cities (provinces/countries). The principal idea of the SCM is based on the "comparative case study", where the effectiveness of an intervention can be identified by comparing the movement of the outcome variable between a treatment group (e.g., cities exposed to intervention) and a control group (similar cities to the treatment group but not exposed to intervention). A weighted average combination of a set of control units is used to construct the counterfactual scenario of the treatment group if it were not exposed to the intervention. Suppose the "synthetic unit" can closely mimic the evolution of the outcome variable for the treatment unit before the policy intervention. In this case, the difference (after the intervention) of outcome movement between treatment unit and synthetic unit can be regarded as the causal impact of the intervention.[1–3]

For the implementation, the goal of SCM is to find a $(J \times 1)$ weights vector $\boldsymbol{W}$ that minimizes the distance between the treatment city and the synthetic city during the pre-treatment periods, where J is the number of control cities. Therefore, the problem can be converted to find the solution to

$\min_{\boldsymbol{W}\ s.t. \sum w_j = 1} \left\| \boldsymbol{V}^{\frac{1}{2}}(\boldsymbol{X}_1 - \boldsymbol{X}'_0\boldsymbol{W}) \right\|$ [4]

where $\left\| \boldsymbol{V}^{\frac{1}{2}}(\boldsymbol{X}_1 - \boldsymbol{X}'_0\boldsymbol{W}) \right\| = \sqrt{(\boldsymbol{X}_1 - \boldsymbol{X}'_0\boldsymbol{W})'\boldsymbol{V}(\boldsymbol{X}_1 - \boldsymbol{X}'_0\boldsymbol{W})}$ is the Euclidean norm of vector for differences between the treatment city and the synthetic city after applying $\boldsymbol{V}$. w_j represents the element in weights vector, $\boldsymbol{V}$ is a symmetric and positive semidefinite matrix (generally defined as an identity matrix in practice) as the importance matrix, $\boldsymbol{X}_1$ is the pre-treatment outcomes (air pollutant concentrations) vector for treatment city, and $\boldsymbol{X}_0$ is the pre-treatment outcomes matrix for control cities. Then, we define the solution above as the SCM weights $\boldsymbol{W}^{scm}$.

Since there is always the possibility for extrapolation from the convex hull of the control units in the real world, leading to minus or greater-than-one values for weights, ASCM employs a penalty function to control the extrapolation to avoid overfitting to errors. Then, ASCM equals to an alternative version of SCM, which is to solve the constrained optimization problem:

$\min_{\boldsymbol{W}\ s.t. \sum w_j = 1} \left\| \boldsymbol{V}^{\frac{1}{2}}(\boldsymbol{X}_1 - \boldsymbol{X}'_0\boldsymbol{W}) \right\|^2 + \zeta \sum f(w_j)$ [5]

where $f(w_j) = (w_j - \hat{w}_j^{scm})^2$ is the penalty function in terms of weights, and the solution can be defined as the Augmented SCM weights $\boldsymbol{W}^{aug}$.

To be specific, the estimated weight $\hat{w}_j^{aug}$ in $\boldsymbol{W}^{aug}$ based on Ridge regression can be calculated through

$\hat{w}_j^{aug} = \hat{w}_j^{scm} + (\boldsymbol{X}_1 - \boldsymbol{X}'_0\boldsymbol{W}^{scm})'(\boldsymbol{X}'_0\boldsymbol{X}_0 + \lambda^{ridge} I_{T_0})^{-1}\boldsymbol{X}_j$. [6]

In addition, $\boldsymbol{W}^{aug}$ is also the solution to

$\min_{\boldsymbol{W}\ s.t. \sum w_j = 1} \|(\boldsymbol{X}_1 - \boldsymbol{X}'_0\boldsymbol{W})\|^2 + \lambda^{ridge} \|\boldsymbol{W} - \boldsymbol{W}^{scm}\|^2$ [7]

where λ^{ridge} is the penalty hyperparameter in the Ridge regression.

Text S2. Limitations of using ML-ASCM.
This section provides further clarifications regarding the synthetic control method and explains how one can apply them in practice[3], especially in air pollution analysis.
a) Choice of control group and spillover effects. In an ideal world, the control and treatment groups have similar characteristics except the interventions in the treatment groups. However, the most important requirement for selecting comparable units as control group is to make sure that no similar interventions are implemented in control units during the whole study period ("no interference", i.e., units within control group should not be affected by any interventions that are similar to the main intervention)[3].

It is not always easy to find suitable control units in air pollution analysis, as air pollution trend are affected by a number of factors (natural variation, socio-economic factors, other policies). Therefore, in practice, it is advisable to remove the units (from control group) that are affected by major interventions, or any other similar policies during the study period. Similar reasoning applies to the potential "spillover effects", where units (for example neighboring cities to the treatment city) that are potentially affected by the spillover effects should also be removed from control group. In addition, units that experience abnormal and large shock in their air quality trends during the study period should be removed as such shocks will lead to large uncertainty in the final estimation.

We emphasize that as long as the synthetic control model is satisfyingly reproducing the pre-intervention observations, the dissimilarity in the characteristics of the control vs. treatment groups should have limited impact on the outcomes.

b) Anticipation. In air pollution control policy evaluations, units within the treatment group might react to the intervention in advance. This will lead to estimation bias if researchers use the "official" policy implementation date, as the true effect will have occurred prior to this date. In this case the researchers are advised to move back the intervention to a date such that the anticipation effect can be fully captured. Using deweathering, a sudden change is often seen in at least some of the pollutants, such as NO2, which often relate to information on the date of any intervention. For winter heating, sudden changes in SO_2 should indicate the heating switch-on.

c) De-noise of the outcome variable. Abadie (2021)[3] pointed out that if the variable of interest is highly volatile, it will be difficult for the synthetic control method to detect the "true" intervention effect, as the noise embedded in the time series trend will confuse the policy effect. This is especially true in air quality trend data, where meteorological conditions add "noise" to the emission data and then complicating the policy evaluation. Abadie (2021)[3] further advise that such noise should be removed before applying the synthetic control method: Our application of the weather normalization technique is essentially in line with this advice. Therefore, future application of the (A)SCM in air quality research should appropriately control / remove the noise, especially from the meteorological variations.

Text S3. $PM_{2.5}$-related health effects from winter heating. The $PM_{2.5}$ exposure-related health effects from winter heating were estimated using the newly developed Global Exposure Mortality

Model (GEMM). The GEMM framework generates a $PM_{2.5}$-mortality relative risk (RR) function based on cohort studies of outdoor air pollution that covers the global exposure range, including high concentrations in China,[4,5] and has been applied in recent studies.[6,7] The GEMM addressed the potential underestimation of mortality at high concentrations using the Integrated Exposure – Response model (IER) in China.[5,8] The GEMM constructed $PM_{2.5}$ concentration-mortality associations for noncommunicable diseases and lower respiratory infections (NCD+LRI), ischemic heart disease (IHD), stroke, chronic obstructive pulmonary disease (COPD), lung cancer (LC), and LRI among adults (≥25 years old). The RR is calculated by the following equation:

$$RR(c) = e^{\frac{\theta \times \log\left(\frac{z}{\alpha}+1\right)}{1+e^{-\frac{z-\mu}{v}}}}, where\ z = \max(0, c - 2.4\ \mu g\ m^{-3})\ [4]$$

where c is long-term ambient $PM_{2.5}$ concentration, e is natural logarithm; θ, α, μ and v are parameters that determine the shape of RR in GEMM and can be found in Table S2 of a previous study.[5] 2.4 μg m^{-3} is the $PM_{2.5}$ threshold below which no effect occurs in the GEMM.

We used the attributable fraction (AF) for diseases to quantify the health effects from winter heating:

$$AF_{yr} = \frac{1}{RR_{counterfactual,yr}} - \frac{1}{RR_{factual,yr}}\ [5]$$

where $RR_{factual,yr}$ and $RR_{counterfactual,yr}$ are RR values for factual and counterfactual annual average $PM_{2.5}$ concentrations in year yr. The estimated AF denotes the fraction of $PM_{2.5}$-related premature deaths from winter heating in all premature deaths caused by NCD+LRI. Premature deaths could be avoided in the counterfactual scenarios can then be estimated by multiplying AF by deaths caused by NCD+LRI from 2019 Global Burden of Disease (GBD) study.[9]

The causal impacts of winter heating on annual average $PM_{2.5}$ concentrations allow us to estimate the corresponding long-term health effects. In this study, the attributable fraction (AF) for $PM_{2.5}$ exposure-associated diseases resulting from winter heating were estimated using a newly developed GEMM with the inclusion of Chinese Cohort.[4,5] We used the annual average of counterfactual deweathered $PM_{2.5}$ concentrations as a baseline to estimate AF for different diseases attributable to winter heating (**Fig. S9**). Winter heating related $PM_{2.5}$ concentrations during 2015-2021 accounted for 2.7 ± 0.3%, 2.6 ± 0.8%, 2.8 ± 0.4% and 1.6 ± 0.4% of premature deaths from noncommunicable diseases and lower respiratory infections (NCD+LRI) deaths in “2+26” cities, “northern” cities, northern and mainland China, respectively. The highest and lowest AFs for NCD+LRI in mainland China were observed in 2020 (~2.3%) and 2018 (~1.1%), corresponding to ~219,261 and ~111,795 premature deaths, respectively. The AF for NCD+LRI in mainland China dropped from ~1.7% in 2015 to ~1.5% in 2021, corresponding to ~169,016 and ~145,460 premature deaths, respectively. The AF values for the other diseases show similar interannual variations with those for NCD+LRI but with different magnitudes (**Fig. S9**).

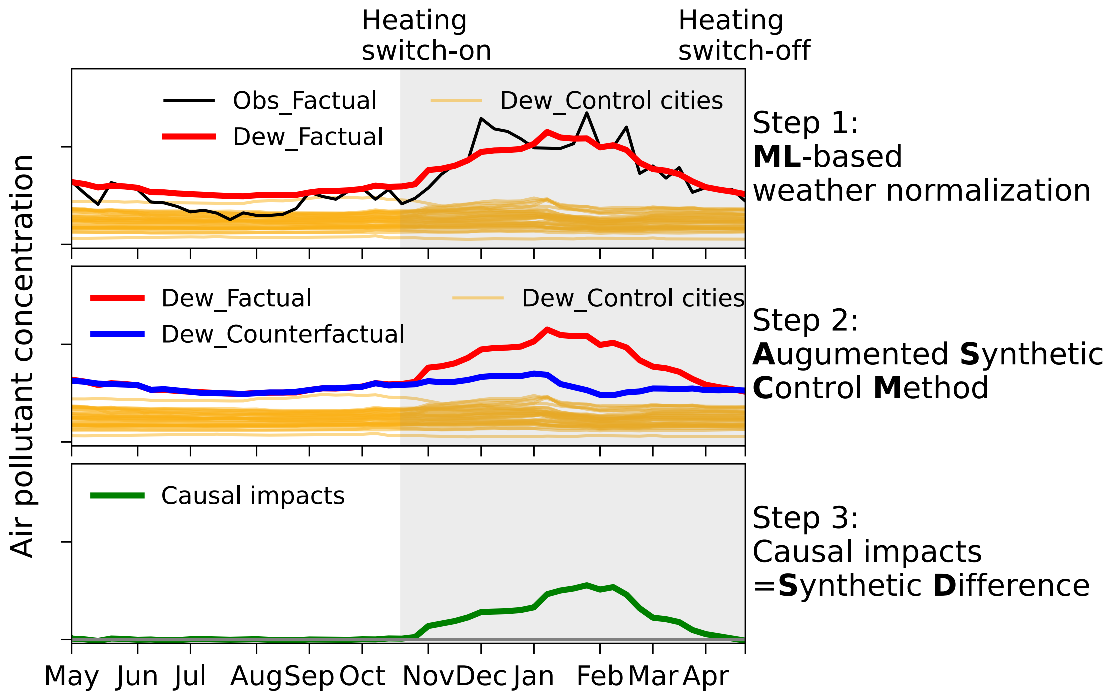

Fig. S1. Concept for estimating the causal impacts. Step1: Apply the "weather normalisation" method to decouple the effects of meteorology from observed air pollutant concentrations (Obs_Factual) to get "deweathered" concentrations (Dew_Factual: weather normalised concentration trend in treatment group where the unit is exposed to the winter heating policy). Step2: Under the "quasi-experimental design", apply the synthetic control approach and use a weighted average of a set of control units (Dew_Control cities: not exposed to winter heating) to construct the counterfactual trend (Dew_Counterfactual) of Dew_Factual if it were not exposed to winter heating. Step3: the difference between Dew_Factual and Dew_Counterfactual during the heating season refers to the causal impact of the winter heating.

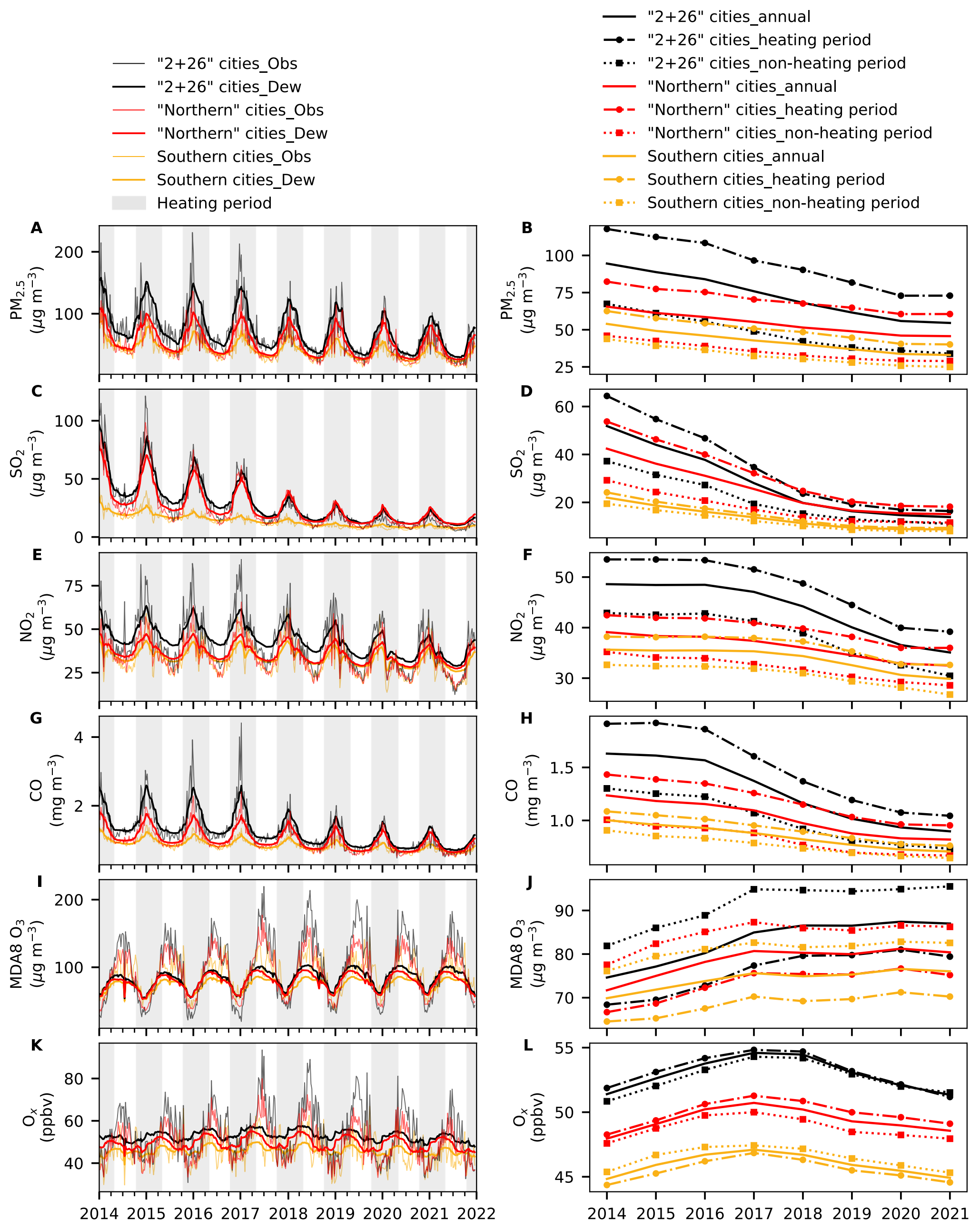

Fig. S2. Air pollutant concentrations in "2+26" cities, "northern" cities, and southern cities. The subfigures in the left panel are observed and deweathered $PM_{2.5}$, SO_2, NO_2, CO, MDA8 O_3, and O_x concentrations in "2+26" cities (in black), "northern" cities (in red), and southern cities (in yellow) at a weekly time resolution from January 2014 to December 2021. The shaded area in grey denotes the heating period. The subfigures in the right panel are interannual variations of deweathered air pollutant concentrations in "2+26" cities, "northern" cities, and southern cities during the heating period (dash-dot lines), the non-heating period (dotted lines) and the whole year (solid lines).

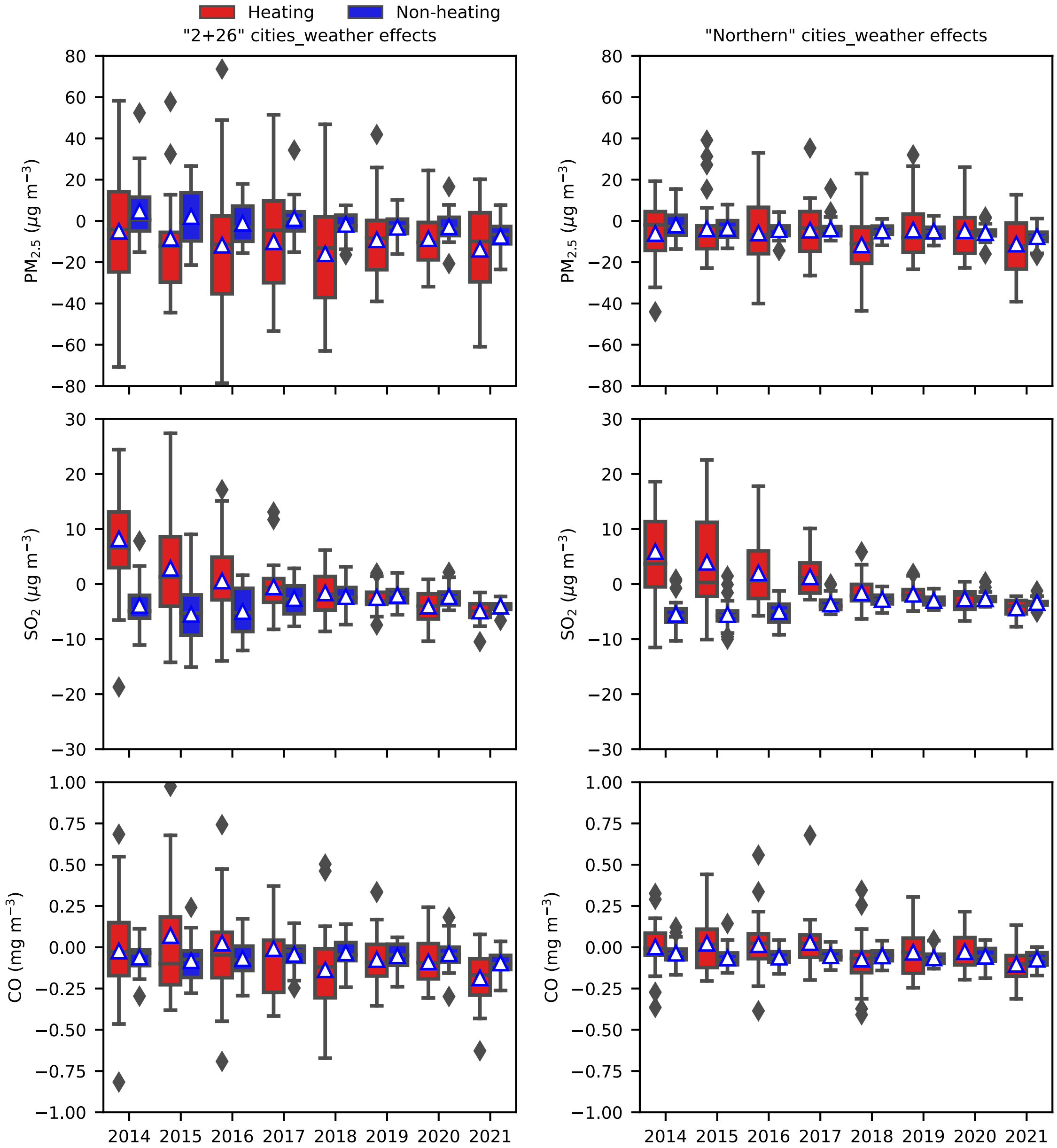

Fig. S3. Boxplots of weather effects on weekly air pollutant concentrations during 2014-2021. We estimated the weather effects on air pollutant concentrations by subtracting weather-normalized concentrations from observed concentrations.

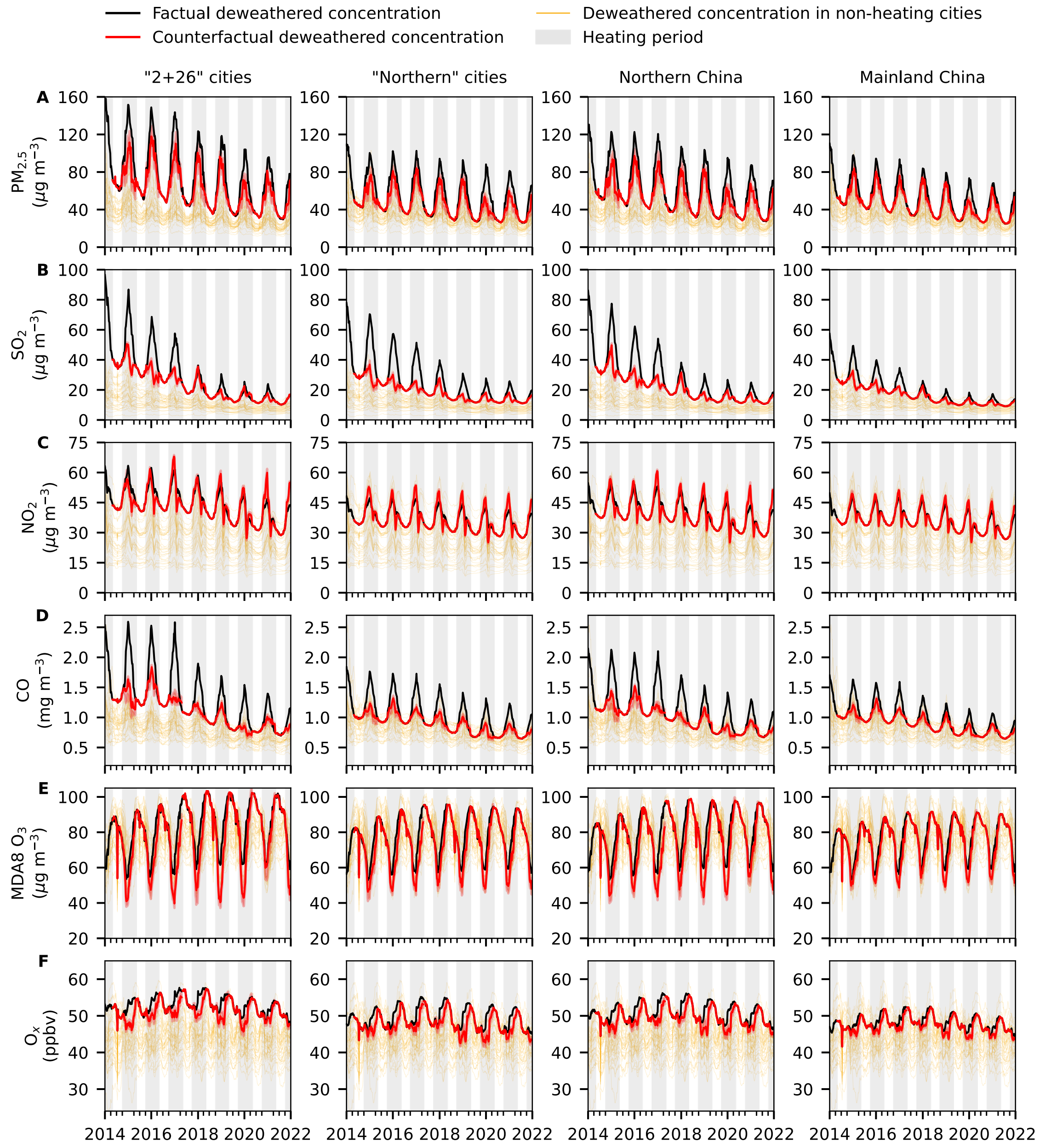

Fig. S4. Factual and counterfactual deweathered concentrations in “2+26” cities, “northern” cities, northern and mainland China, and deweathered concentrations in non-heating cities (in yellow).

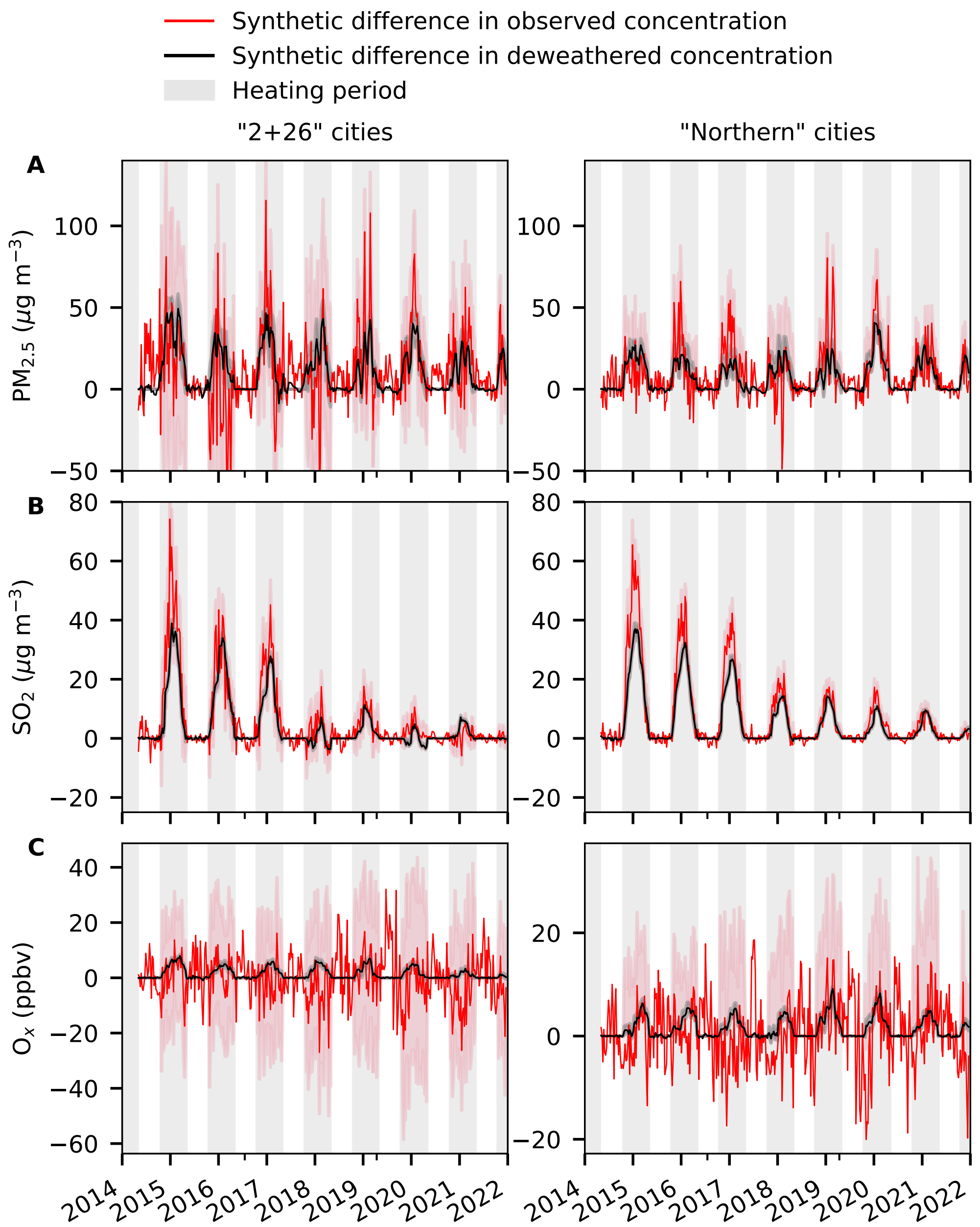

Fig. S5. Synthetic difference in observed weekly air pollutant concentration (in red) and synthetic difference in deweathered weekly air pollutant concentration (in black) for PM2.5, SO2 and Ox. The shaded area of the effect from Ridge ASCM for "2+26" cities and "northern" cities denotes the pointwise 95% confidence interval using Jackknife+ procedure.

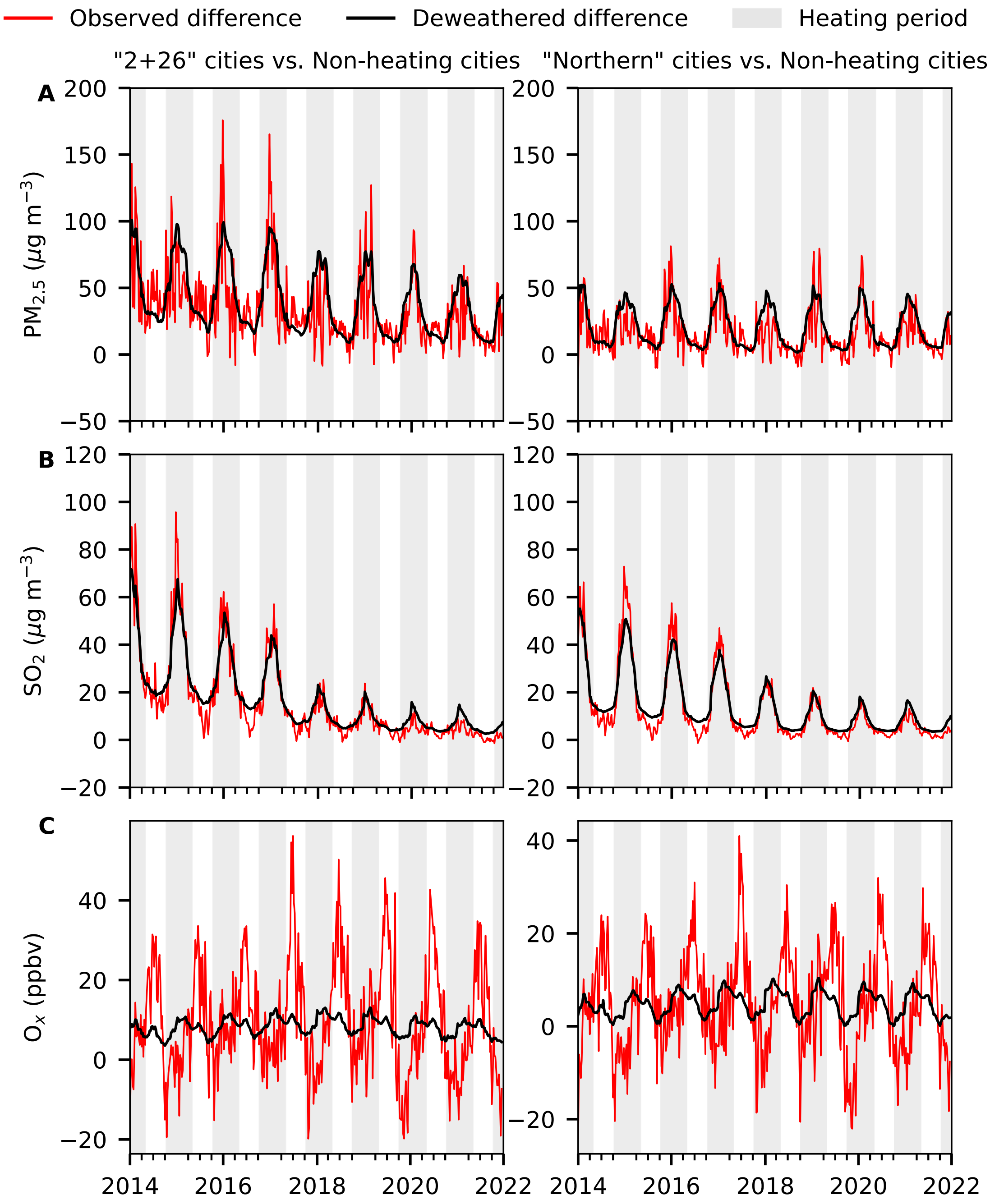

Fig. S6. Observed difference and deweathered difference in weekly air pollutant concentrations between “2+26” cities/ “northern” cities and non-heating cities.

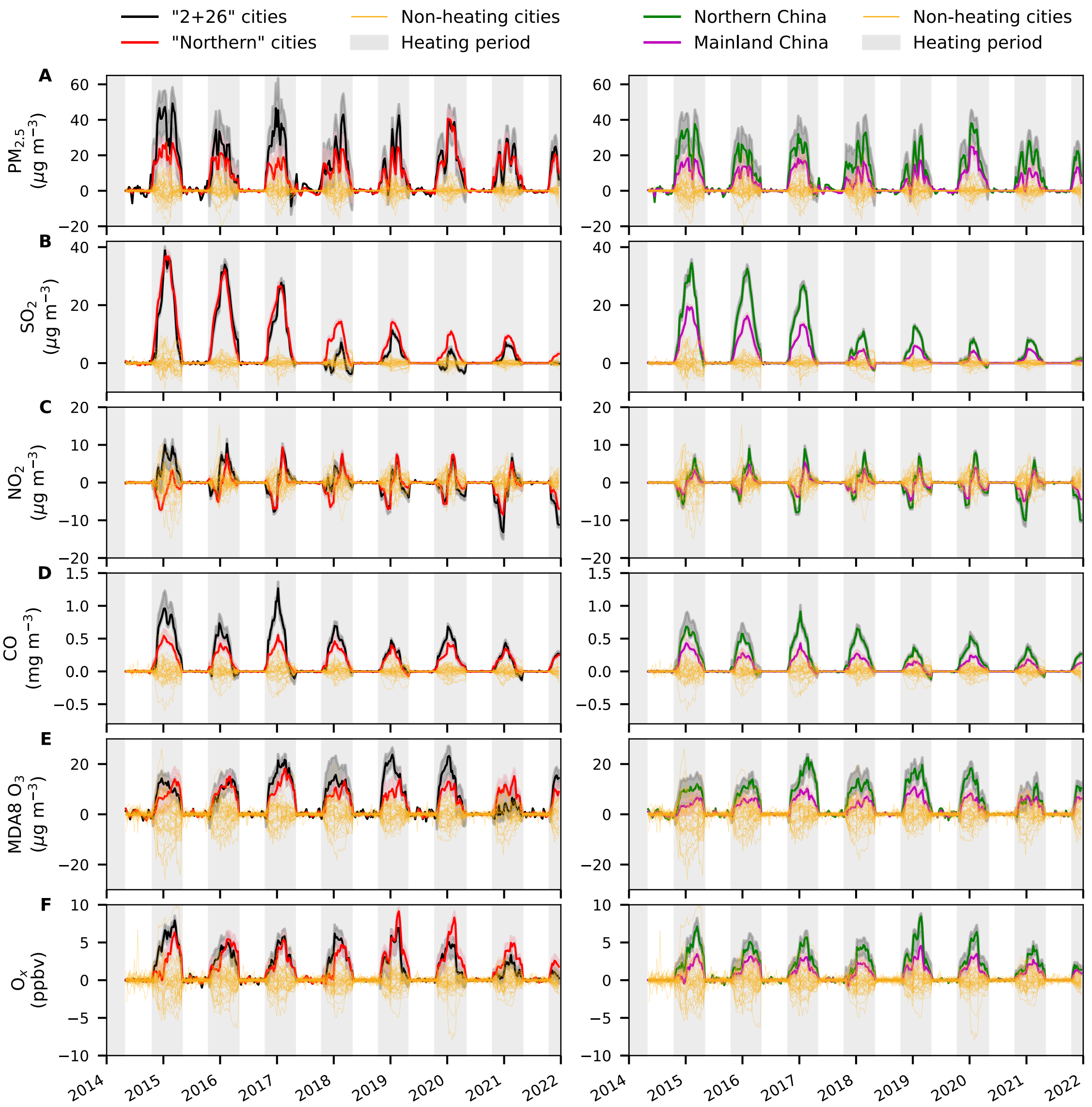

Fig. S7. Synthetic difference in deweathered weekly air pollutant concentrations in "2+26" cities, "northern" cities, northern China, and mainland China. The results for "2+26" cities and "northern" cities are shown in the left panel whereas the results for northern and mainland China are shown in the right panel. The in-place placebo tests for each city in the control group are shown in yellow. The shaded area of the effect from ridge ASCM denotes the pointwise 95% confidence interval using Jackknife+ procedure. The shaded area in grey denotes the heating period.

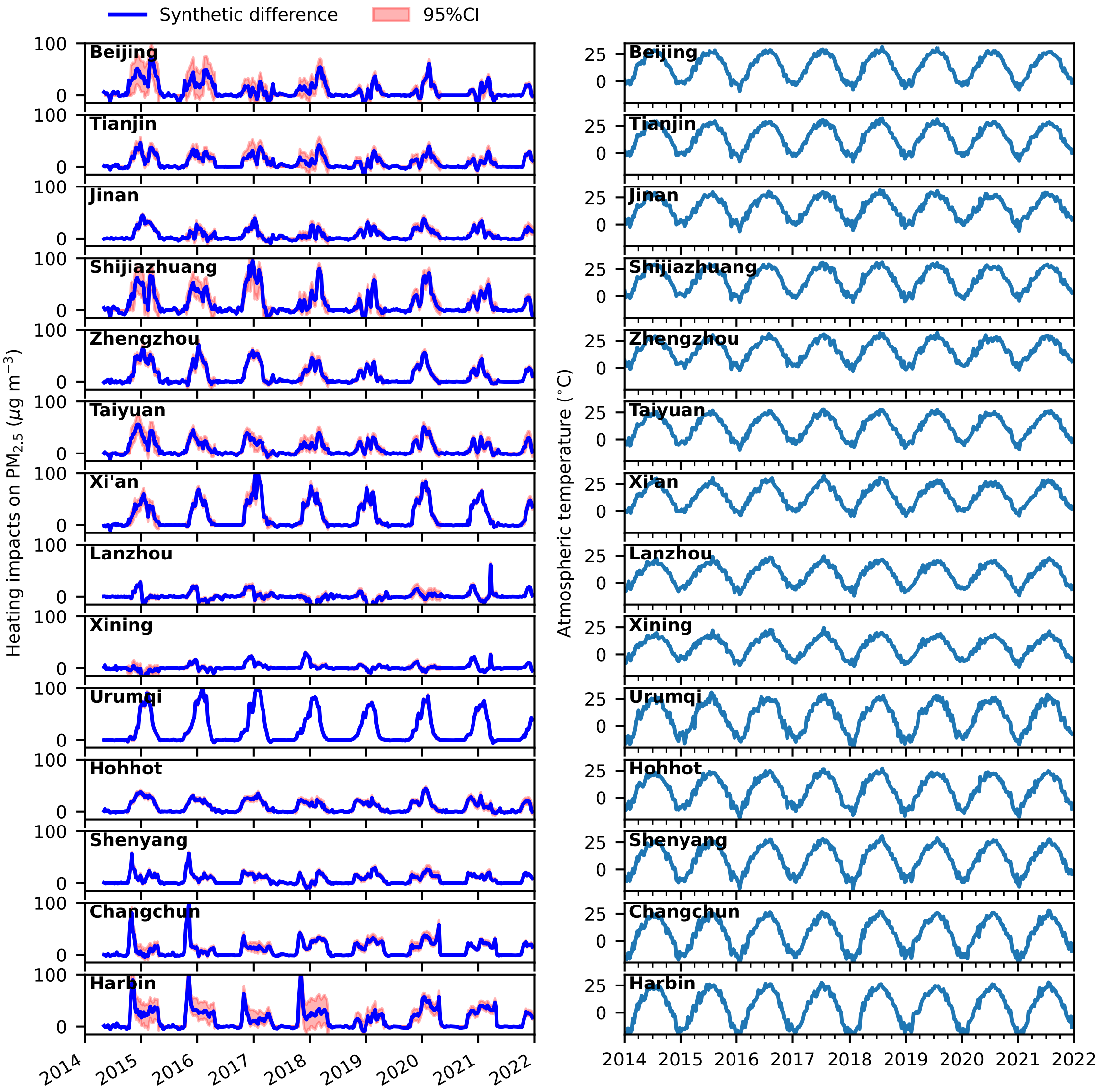

Fig. S8. Time series of synthetic difference (with 95% confidence interval) in deweathered weekly $PM_{2.5}$ concentrations (left panel) in the 14 major northern cities and weekly atmospheric temperature (right panel) in the 14 major northern cities. Lanzhou: Implementation of "double zeroing" of coal-fired boilers in urban areas by 2014 http://fgcx.bjcourt.gov.cn:4601/law?fn=lar806s131.txt&truetag=974&titles=&contents=&dbt=lar (accessed Dec 31, 2022)
Xining: Xining completed “coal to gas” projects ahead of the other northern Chinese cities. http://covid-19.chinadaily.com.cn/hqgj/jryw/2016-02-25/content_14569618.html (accessed Dec 31, 2022)
Jinan: Jinan takes the lead in "coal to gas" for coal-fired boilers during 2013-2015. http://sd.ifeng.com/news/chengshi/detail_2013_12/04/1548086_0.shtml (accessed Dec 31, 2022).
Note that large interannual variations (**Table S7**) in the synthetic differences in deweathered $PM_{2.5}$ were observed in some of the northern cities. This reflects the impacts of different control measures on winter heating emissions in different years rather than varying heating demands in different years or high model uncertainties. This is supported by low interannual variations in the atmospheric temperature and low 95% confidence intervals in the synthetic differences (**Fig. S8**).

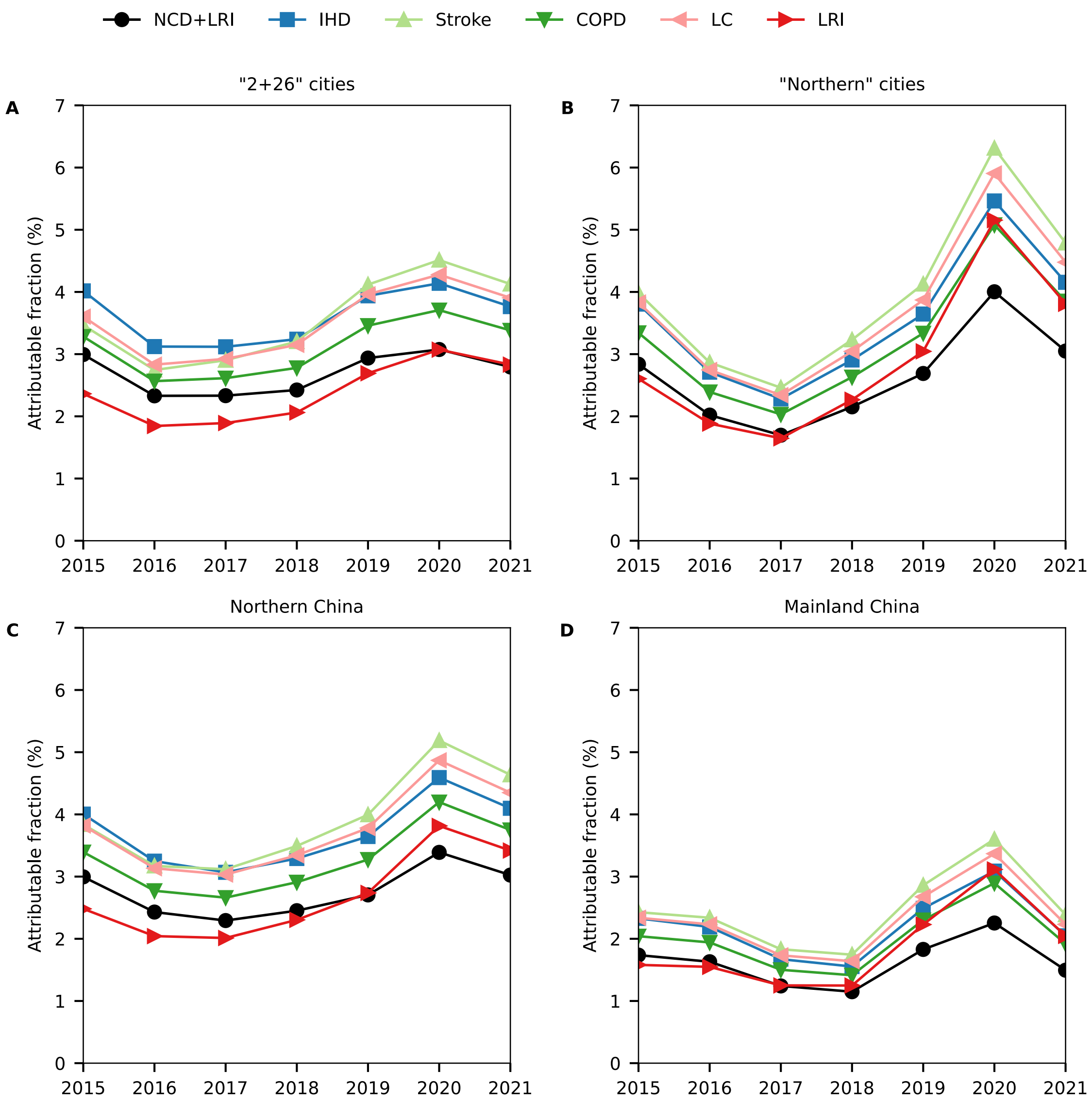

Fig. S9. Attributable fractions for different diseases as a result of the higher PM2.5 concentrations from winter heating in “2+26” cities, “northern” cities, northern and mainland China from 2015 to 2021.

Table S1. Classification of the 189 Chinese cities studied.

	Province	Cities
Northern China (90 cities)	***"2+26" cities (24 cities) – treatment group***	
	Beijing	Beijing
	Tianjin	Tianjin
	Shandong	Dezhou, Jinan, Zibo, Binzhou, Liaocheng, Heze, Jinning
	Shanxi	Taiyuan, Changzhi, Yangquan
	Hebei	Baoding, Tangshan, Langfang, Cangzhou, Shijiazhuang, Hengshui, Xingtai, Handan
	Henan	Zhengzhou, Anyang, Kaifeng, Jiaozuo
	"Northern" cities (66 cities)- treatment group	
	Gansu	Lanzhou, Jiayuguan, Jinchang
	Hebei	Zhangjiakou, Chengde, Qinhuangdao
	Heilongjiang	Harbin, Mudanjiang, Qiqihar, Daqing
	Henan	Sanmenxia, Pingdingshan, Luoyang
	Inner Mongolia	Baotou, Hohhot, Ordos, Chifeng
	Jilin	Changchun, Jilin
	Liaoning	Dandong, Dalian, Shenyang, Panjin, Yingkou, Huludao, Fushun, Benxi, Wafangdian, Jinzhou, Anshan
	Ningxia	Yinchuan, Shizuishan
	Qinghai	Xining
	Shaanxi	Xianyang, Baoji, Yanan, Weinan, Xi'an, Tongchuan
	Shandong	Dongying, Linyi, Weihai, Rizhao, Zaozhuang, Taian, Weifang, Yantai, Qingdao, Rushan, Jimo, Shouguang, Pingdu, Zhaoyuan, Wendeng, Zhangqiu, Jiaonan, Jiaozhou, Rongcheng, Laizhou, Laixi, Penglai
	Shanxi	Linfen, Datong
	Xinjiang	Urumqi, Karamay, Kuerle
Southern China (99 cities)	***Alternative cities (9 cities) – treatment group***	
	Tibet	Lhasa
	Anhui	Hefei
	Yunnan	Kunming, Qujing, Yuxi
	Hunan	Changsha, Xiangtan, Zhuzhou
	Hubei	Wuhan
	Non-heating cities (39 cities) – control group	
	Fujian	Xiamen, Quanzhou, Fuzhou
	Guangdong	Dongguan, Zhongshan, Foshan, Guangzhou, Huizhou, Shantou, Jiangmen, Heyuan, Shenzhen, Zhuhai, Zhaoqing, Shaoguan, Jieyang, Meizhou, Shanwei, Zhanjiang, Chaozhou, Maoming, Yangjiang
	Guangxi	Beihai, Nanning, Liuzhou
	Haiyang	Haikou, Sanya
	Jiangxi	Nanchang
	Zhejiang	Lishui, Taizhou, Jiaxing, Ningbo, Hangzhou, Wenzhou, Huzhou, Shaoxing, Zhoushan, Quzhou, Jinhua
	Other southern cities (51 cities)	
	Anhui	Wuhu, Maanshan
	Chongqing	Chongqing
	Guangdong	Qingyuan, Yunfu
	Guangxi	Guilin
	Guizhou	Guiyang, Zunyi
	Hubei	Yichangn, Jingzhou
	Hunan	Yueyang, Changde, Zhangjiajie
	Jiangsu	Nanjing, Nantong, Suqian, Changzhou, Xuzhou, Yangzhou, Wuxi, Taizhou, Huaian, Yancheng, Suzhou, Lianyungang, Zhenjiang, Jurong, Wujiang, Taicang, Yixing, Fuyang, Changshu, Zhangjiagang, Kunshan, Jiangyin, Haimen, Liyang, Jintan
	Jiangxi	Jiujiang
	Shanghai	Shanghai
	Sichuan	Chengdu, Nanchong, Yibin, Deyang, Panzhihua, Luzhou, Mianyang, Zigong
	Zhejiang	Linan, Yiwu, Zhuji

Table S2. List of winter heating start dates.

Starting date	Cities
9-Oct	Urumqi, Daqing
13-Oct	Qiqihar
14-Oct	Baotou, Hohhot, Zhangjiakou, Xining, Ordos, Chifeng
20-Oct	Harbin
24-Oct	Datong, Changchun, Jilin
26-Oct	Shizuishan
27-Oct	Chengde
29-Oct	Panjin
31-Oct	Linfen, Dandong, Lanzhou, Taiyuan, Shenyang, Yingkou, Huludao, Yinchuan, Yangquan, Jiayuguan, Fushun, Benxi, Jincheng, Jinzhou, Anshan
1-Nov	Yanan, Kuerle
4-Nov	Dalian, Wafangdian
5-Nov	Qinhuangdao
6-Nov	Weihai, Rongcheng
9-Nov	Linyi, Taian, Jining, Hengshui
10-Nov	Shouguang
11-Nov	Jinan, Liaocheng, Zhangqiu
14-Nov	Weinan, Zhengzhou, Sanmenxia, Karamay, Anyang, Kaifeng, Luoyang, Jiaozuo, Mudanjiang
15-Nov	Dongying, Baoding, Beijing, Tangshan, Tianjin, Baoji, Langfang, Cangzhou, Zibo, Binzhou, Weifang, Yantai, Shijiazhuang, Heze, Xian, Xingtai, Handan, Changzhi, Qingdao, Pingdingshan, Xianyang, Zaozhuang, Tongchuan, Jimo, Pingdu, Zhaoyuan, Jiaonan, Jiaozhou, Laizhou, Laixi
20-Nov	Wendeng
24-Nov	Rizhao, Rushan

Sources: Online information from local governments and from Fan et al.[10]

Table S3. Model performance parameters for the testing data set of each air pollutant (in hourly time resolution) in 2016-2020. ($PM_{2.5}$: μg m^{-3}, NO_2: μg m^{-3}, SO_2: μg m^{-3}, CO: mg m^{-3}, O_3_8h: μg m^{-3}, O_x: ppbv)

	"2+26" cities	"Northern" citie	Non-heating cities	Alternative cities	Other southern cities
**Mean bias**					
$PM_{2.5}$	-0.53±0.44	-0.64±0.39	-0.25±0.22	-0.4±0.29	-0.39±0.38
NO_2	-0.36±0.23	-0.23±0.14	-0.15±0.13	-0.16±0.18	-0.2±0.17
SO_2	-0.36±0.33	-0.42±0.41	-0.13±0.14	-0.23±0.18	-0.19±0.17
CO	-0.01±0.01	-0.01±0.01	-0.0±0.0	-0.0±0.01	-0.0±0.0
O_3_8h	-0.16±0.3	-0.23±0.32	-0.27±0.25	-0.27±0.31	-0.19±0.29
O_x	-0.16±0.22	-0.14±0.15	-0.17±0.14	-0.12±0.11	-0.12±0.19
**Normalized mean bias**					
$PM_{2.5}$	-0.01±0.01	-0.01±0.01	-0.01±0.01	-0.01±0.01	-0.01±0.01
NO_2	-0.01±0.01	-0.01±0.0	-0.01±0.0	-0.01±0.01	-0.01±0.01
SO_2	-0.01±0.01	-0.02±0.01	-0.01±0.01	-0.02±0.01	-0.01±0.01
CO	-0.01±0.01	-0.01±0.01	-0.0±0.0	-0.01±0.01	-0.0±0.0
O_3_8h	-0.0±0.0	-0.0±0.0	-0.0±0.0	-0.01±0.01	-0.0±0.01
O_x	-0.0±0.0	-0.0±0.0	-0.0±0.0	-0.0±0.0	-0.0±0.0
**Normalized mean gross error**					
$PM_{2.5}$	0.33±0.03	0.36±0.05	0.29±0.03	0.27±0.02	0.31±0.04
NO_2	0.24±0.02	0.25±0.04	0.24±0.03	0.22±0.03	0.24±0.03
SO_2	0.34±0.03	0.33±0.08	0.25±0.06	0.26±0.07	0.29±0.05
CO	0.25±0.06	0.24±0.05	0.14±0.02	0.18±0.04	0.18±0.04
O_3_8h	0.23±0.02	0.2±0.03	0.24±0.03	0.21±0.03	0.23±0.03
O_x	0.13±0.01	0.13±0.02	0.17±0.02	0.13±0.02	0.15±0.02
**Correlation coefficient**					
$PM_{2.5}$	0.82±0.03	0.78±0.07	0.81±0.03	0.83±0.04	0.8±0.05
NO_2	0.84±0.02	0.83±0.03	0.83±0.03	0.85±0.05	0.82±0.03
SO_2	0.84±0.04	0.82±0.08	0.72±0.11	0.77±0.1	0.78±0.09
CO	0.82±0.05	0.81±0.09	0.81±0.08	0.81±0.04	0.8±0.07
O_3_8h	0.92±0.01	0.89±0.02	0.85±0.02	0.9±0.02	0.88±0.02
O_x	0.89±0.01	0.87±0.03	0.85±0.02	0.89±0.02	0.87±0.03

Table S4. Model performance of the Ridge ASCM on **deweathered** concentrations. Scaled imbalance from non-heating period outcomes by ridge ASCM and percent improvement in bias during non-heating periods using ASCM compared to those using uniform weights.

city	year	Scaled imbalance during non-heating periods						Improvement (%) from uniform weights					
		$PM_{2.5}$ (μg m^{-3})	SO_2 (μg m^{-3})	NO_2 (μg m^{-3})	CO (mg m^{-3})	MDA8 O_3 (μg m^{-3})	O_x (ppbv)	$PM_{2.5}$	SO_2	NO_2	CO	MDA8 O_3	O_x
**"2+26" cities**	**2015**	0.1	0	0	0	0.1	0	92.6	97.9	99.2	98.3	92	95.1
	**2016**	0	0	0	0	0.1	0	99.4	98.2	99.5	99.6	93.3	96.8
	**2017**	0.2	0	0	0	0.1	0	81.8	99.4	99.1	96.4	92.6	98.4
	**2018**	0.1	0	0	0	0.1	0	93.9	99	98.7	97.1	93.8	99.1
	**2019**	0.1	0	0	0.1	0.1	0	85.4	99.9	97.1	88.8	90.2	98.4
	**2020**	0	0	0	0	0	0	95.7	98.5	98.8	99.6	97.8	98.7
	**2021**	0.1	0	0	0.1	0.1	0	92	98.5	97.8	94.6	93.4	98.4
**"Northern" cities**	**2015**	0.1	0	0	0.1	0.1	0.1	90.8	98.7	97.5	92.6	89.9	94.2
	**2016**	0.1	0	0	0.1	0	0	93.7	97.5	99.6	92	96.3	96.2
	**2017**	0.3	0	0	0.1	0.1	0	67.7	99.1	97.9	90.1	93.3	95.1
	**2018**	0.2	0	0	0	0	0	84.4	97	98.3	95.8	96.5	99.2
	**2019**	0.2	0	0	0.5	0.1	0	80.3	99.9	97.1	53.5	89.9	98.4
	**2020**	0.1	0	0	0.2	0	0	91.2	99.6	98.2	84.9	97.8	98.4
	**2021**	0.1	0	0	0.1	0.1	0	90.4	99	98.3	90.7	93.2	96.9
**Northern China**	**2015**	0.1	0	0	0	0.1	0.1	92.3	98.4	99.2	95.6	89.3	95
	**2016**	0	0	0	0	0	0	96.7	98	99.7	99.5	97.6	96.8
	**2017**	0.2	0	0	0.1	0.1	0	75.7	99.6	98.6	92.9	93.1	97.2
	**2018**	0.1	0	0	0	0	0	89.2	98.8	98.8	99.7	96.9	99.5
	**2019**	0.1	0	0	0.2	0.1	0	86	99.9	98.5	81.3	88.9	97.5
	**2020**	0.1	0	0	0	0	0	93	99.2	99	96	97.8	98.4
	**2021**	0.1	0	0	0.1	0.1	0	91.3	99.3	98.3	93.5	94.2	99.9
**Mainland China**	**2015**	0.1	0	0	0	0.1	0	93.6	98.7	99.2	95.8	87.3	96.3
	**2016**	0	0	0	0	0.1	0	96.2	98.2	99.7	99	95	97.6
	**2017**	0.2	0	0	0.1	0	0	82.5	99.6	98.7	94.8	95.6	97
	**2018**	0.1	0	0	0	0.1	0	93.7	98.8	99.1	99.9	94.6	99.8
	**2019**	0.2	0	0	0.1	0.1	0	84.3	99.7	99.3	89.8	89.7	98.6
	**2020**	0	0	0	0	0	0	97.9	99.1	99.6	97.1	96.8	98.7
	**2021**	0	0	0	0.1	0.1	0	95.1	99.3	98.9	94.9	94	99.6

Table S5. Model performance of the Ridge ASCM on **observed** concentrations. Scaled imbalance from non-heating period outcomes by ridge ASCM and percent improvement in bias during non-heating periods using ASCM compared to those using uniform weights.

city	year	Scaled imbalance during non-heating periods						Improvement (%) from uniform weights					
		$PM_{2.5}$ (μg m^{-3})	SO_2 (μg m^{-3})	NO_2 (μg m^{-3})	CO (mg m^{-3})	MDA8 O_3 (μg m^{-3})	O_x (ppbv)	$PM_{2.5}$	SO_2	NO_2	CO	MDA8 O_3	O_x
**"2+26" cities**	**2015**	0.1	0.1	0.2	0.4	0.6	0.4	89.9	94.1	75.6	58.7	38.6	57.2
	**2016**	0.3	0.3	0.1	0.1	0.7	0.3	66.7	68.5	86	90.2	31.2	65.6
	**2017**	0.6	0.3	0.3	0.3	0.5	0.2	37.5	71.9	67.3	68.2	45.2	78
	**2018**	0.5	0.4	0.3	0.4	0.6	0.4	53.1	55.4	74.9	64.6	42.5	56.7
	**2019**	0.7	0.3	0.3	0.4	0.6	0.6	32.2	71.5	72	60.4	37.1	43.1
	**2020**	0.6	0.3	0.2	0.5	0.6	0.3	39.4	66.2	84.5	53.2	44.9	70.4
	**2021**	0.5	0.6	0.4	0.6	0.6	0.4	45	44.3	56.9	42.8	35.9	56.9
**"Northern" cities**	**2015**	0.5	0.2	0.2	0.6	0.5	0.4	54.1	80.2	84	44.2	46.9	58.6
	**2016**	0.6	0.3	0.3	0	0.6	0.4	40.4	66.6	74.1	98.8	42.2	56.3
	**2017**	0.8	0.3	0.4	0.5	0.5	0.4	24.7	68.7	58.1	53	48.9	57.4
	**2018**	0.5	0.4	0.2	0.6	0.6	0.5	45.2	61.8	77.1	41.7	43.1	53.1
	**2019**	0.8	0.4	0.3	0.7	0.6	0.6	24.1	63.2	66.7	26.5	39.3	43.7
	**2020**	0.5	0.4	0.3	0.6	0.5	0.4	49.3	63.3	66.8	39	46.5	55.5
	**2021**	0.5	0.4	0.2	0.6	0.6	0.3	46.2	64.5	79.1	44.2	38.2	66.5
**Northern China**	**2015**	0	0	0.2	0.4	0.6	0.4	97.3	96.7	81.8	56.8	43.3	57.5
	**2016**	0.4	0.3	0.1	0.1	0.6	0.4	64.4	69.2	90.6	93.8	36	65
	**2017**	0.7	0.3	0.4	0.4	0.5	0.4	33.4	71.4	62.1	64.5	47.7	57.7
	**2018**	0.5	0.4	0.3	0.4	0.6	0.4	47.3	64.2	74.8	59.6	43.6	57.5
	**2019**	0.7	0.3	0.3	0.5	0.6	0.6	28.9	68.6	71.1	47.8	38.7	43.8
	**2020**	0.6	0.4	0.3	0.5	0.5	0.4	44.6	61.7	73	50.3	48	64.5
	**2021**	0.5	0.4	0.3	0.6	0.6	0.3	47.4	57.2	71.8	45	37.4	65.8
**Mainland China**	**2015**	0	0.1	0.1	0.4	0.4	0.3	98	91.7	88.9	62.9	58.5	67.7
	**2016**	0.3	0.2	0	0	0.5	0.3	66.2	77.8	97.5	98.6	45.8	71.1
	**2017**	0.6	0.2	0.2	0.3	0.4	0.3	42.3	78.9	76.1	74.5	57.8	67.9
	**2018**	0.4	0.3	0.1	0.3	0.5	0.3	61	71.1	87.2	65.4	52.9	68.6
	**2019**	0.6	0.3	0.2	0.5	0.5	0.4	44	68.8	82.2	45.4	52.1	58.1
	**2020**	0.4	0.3	0.2	0.3	0.5	0.3	58.9	74.7	84.4	66.6	53.4	69.8
	**2021**	0.4	0.3	0.1	0.4	0.5	0.3	59.9	66	85.5	63	49.6	72.1

Table S6. Synthetic difference in observed and deweathered air pollutant concentrations.

Year	Observation						Deweathered					
	$PM_{2.5}$ (μg m^{-3})	SO_2 (μg m^{-3})	CO (mg m^{-3})	NO_2 (μg m^{-3})	MDA8 O_3 (μg m^{-3})	O_x (ppbv)	$PM_{2.5}$ (μg m^{-3})	SO_2 (μg m^{-3})	CO (mg m^{-3})	NO_2 (μg m^{-3})	MDA8 O_3 (μg m^{-3})	O_x (ppbv)
**"2+26" cities**												
**2015**	8.2	14.2	0.47	2.4	0.1	2.0	14.3	9.8	0.27	1.6	3.9	2.3
**2016**	5.0	10.9	0.36	0.0	-3.6	0.6	10.6	9.1	0.21	0.6	5.7	1.6
**2017**	13.2	6.5	0.21	-2.4	-4.8	-1.5	9.5	4.5	0.21	0.8	7.6	1.6
**2018**	9.5	2.1	0.07	-2.0	5.2	-0.2	8.8	0.6	0.15	-0.1	6.6	1.8
**2019**	13.7	2.9	0.10	-0.2	2.4	1.9	9.6	1.6	0.13	-0.3	6.8	1.7
**2020**	16.4	1.0	0.13	-0.3	2.1	-0.9	9.3	0.2	0.14	-1.4	3.7	1.0
**2021**	12.3	0.6	0.05	-1.2	2.2	-1.9	8.4	1.2	0.08	-0.9	2.7	0.7
**"Northern" cities**												
**2015**	9.3	15.3	0.19	0.0	-2.3	1.3	9.3	10.8	0.15	-0.6	4.3	1.6
**2016**	9.1	12.1	0.18	-0.4	-2.4	0.3	6.5	8.9	0.13	-0.1	4.7	1.7
**2017**	8.2	9.0	0.10	-1.4	-6.0	-1.0	5.2	6.1	0.13	-0.3	5.6	1.2
**2018**	5.5	4.6	0.08	-1.2	-0.2	1.0	6.3	3.6	0.12	-0.1	4.0	1.6
**2019**	12.1	4.2	0.10	-0.6	0.3	1.2	7.5	3.1	0.09	-0.2	4.1	2.1
**2020**	12.9	3.7	0.12	0.8	1.0	1.8	10.5	2.2	0.11	-0.4	3.6	1.8
**2021**	8.4	2.1	0.08	-1.2	-2.1	0.8	8.1	2.2	0.09	-0.5	4.2	1.5
**Northern China**												
**2015**	5.3	14.5	0.30	0.4	-1.7	1.5	11.5	9.0	0.20	0.3	4.2	1.9
**2016**	6.6	11.3	0.25	-0.4	-3.0	0.4	9.0	8.8	0.16	0.1	4.9	1.6
**2017**	9.6	7.8	0.16	-1.7	-5.8	0.0	7.9	5.4	0.18	0.1	7.4	1.4
**2018**	6.7	3.4	0.08	-1.6	1.9	1.6	7.8	2.2	0.15	0.1	5.6	1.6
**2019**	11.6	3.5	0.10	-0.3	0.7	1.2	8.1	2.4	0.09	-0.3	6.3	1.9
**2020**	14.3	2.5	0.13	0.1	1.4	0.7	9.5	1.3	0.12	-0.8	3.6	1.4
**2021**	10.1	1.5	0.07	-1.6	0.1	-0.2	8.4	1.7	0.08	-0.8	3.5	1.2
**Mainland China**												
**2015**	2.1	7.8	0.15	0.2	-3.0	0.7	5.7	5.4	0.11	0.1	2.1	0.9
**2016**	3.8	5.5	0.13	-0.4	-3.7	0.1	5.1	4.3	0.08	0.1	2.7	1.0
**2017**	4.7	3.5	0.06	-0.9	-5.2	-0.4	3.7	2.7	0.08	0.1	2.8	0.7
**2018**	3.8	1.5	0.03	-0.7	-0.1	0.9	3.3	0.9	0.06	-0.3	2.8	0.8
**2019**	6.4	1.3	0.05	-0.2	0.8	1.0	4.9	1.2	0.04	-0.4	3.1	1.1
**2020**	7.4	0.9	0.07	0.1	0.7	0.5	5.7	0.7	0.06	-0.4	2.1	0.8
**2021**	5.3	0.5	0.03	-1.1	-2.0	-0.4	3.8	1.0	0.04	-0.2	2.3	0.7

Table S7. Heating impacts on annual $PM_{2.5}$ (μg m^{-3}), SO_2 (μg m^{-3}) and CO (mg m^{-3}) in 14 major cities in northern China from 2015 to 2021.

		"2+26" cities						Northwest				Northeast			
	**Year**	**Beijing**	**Tianjin**	**Jinan**	**Shijiazhuang**	**Zhengzhou**	**Taiyuan**	**Xi'an**	**Lanzhou**	**Xining**	**Urumqi**	**Hohhot**	**Shenyang**	**Changchun**	**Harbin**
**$PM_{2.5}$**	**2015**	14.5	10.5	4.4	14.4	12.6	10.0	14.7	0.5	1.4	23.7	9.3	9.0	10.5	18.4
	**2016**	3.4	10.3	5.5	22.0	14.8	9.5	21.8	3.6	4.7	25.1	11.0	7.6	7.9	9.6
	**2017**	12.8	9.6	4.6	12.9	11.9	9.0	17.7	-3.8	4.3	21.7	8.0	3.8	13.9	16.9
	**2018**	3.5	4.4	7.0	5.7	8.3	7.2	15.9	-3.2	0.7	18.3	7.8	8.1	11.8	6.4
	**2019**	7.8	8.0	9.3	15.7	12.1	11.4	20.1	2.2	1.3	18.6	10.4	8.2	13.4	18.4
	**2020**	3.4	5.0	6.7	10.2	7.3	6.5	14.8	2.9	2.5	18.0	7.6	6.9	10.2	17.2
	**2021**	3.3	5.5	4.3	3.2	5.0	4.9	8.3	2.6	1.4	6.4	4.6	4.7	6.0	5.9
**SO_2**	**2015**	4.9	7.0	10	10.7	6.6	28.7	6.2	5.3	3.1	4.1	12.7	20.8	13.1	13.2
	**2016**	4.0	5.3	3.0	10.5	4.8	31.3	5.5	6.2	3.7	3.9	13	15.7	11.4	10.6
	**2017**	2.4	1.5	2.6	3.0	-4.6	18.0	3.1	6.5	0.2	2.2	6.8	3.9	6.9	10.4
	**2018**	1.7	2.6	3.7	3.6	0.9	10.0	1.9	5.3	1.9	0.9	5.4	3.7	4.7	7.2
	**2019**	0.7	0.5	3.7	2.0	0.7	6.8	2.5	3.4	2.5	1.4	5.6	3.8	4.1	6.3
	**2020**	0.8	1.2	3.0	2.2	0.9	5.5	1.7	3.4	3.0	0.5	4.5	2.2	3.6	4.9
	**2021**	0.3	0.2	0.7	0.2	-0.2	3.5	0.5	1.8	0.9	0.3	2.0	1.1	1.3	1.2
**CO**	**2015**	0.27	0.03	0.14	0.30	-0.06	0.09	0.18	0.23	0.25	0.44	0.27	0.09	0.11	0.15
	**2016**	0.18	0.23	0.22	0.39	0.16	0.31	0.26	0.25	0.26	0.42	0.28	0.07	0.18	0.14
	**2017**	0.15	0.15	0.11	0.29	0.11	0.07	0.20	0.30	0.27	0.34	0.24	0.11	0.09	0
	**2018**	0.09	0.09	0.06	0.19	-0.01	0.12	0.06	0.20	0.25	0.23	0.21	0.09	-0.01	0.09
	**2019**	0.12	0.15	0.12	0.20	0.13	0.12	0.12	0.27	0.24	0.27	0.23	0.13	0.12	0.09
	**2020**	0.07	0.08	0.07	0.20	0.04	0.10	0.09	0.15	0.15	0.20	0.19	0.06	0.08	0.07
	**2021**	0.02	0.04	0.03	0.05	0.03	0.04	0.06	0.10	0.09	0.10	0.10	0.04	0.04	0.03

Table S8. Ordinary least squares (OLS) regressions between synthetic difference in $PM_{2.5}$ against other pollutants. ($PM_{2.5}$: μg m^{-3}, NO_2: μg m^{-3}, SO_2: μg m^{-3}, CO: mg m^{-3}, O_x: ppbv)

	Region	Model	R^2	Coefficient of O_x
Observed	"2+26"cities	$PM_{2.5}$=f(O_x)	0.06	0.6
		$PM_{2.5}$=f(O_x, SO_2)	0.12	0.5
		$PM_{2.5}$=f(O_x, NO_2)	0.18	0.3
		$PM_{2.5}$=f(O_x, CO)	0.26	0.5
		$PM_{2.5}$=f(O_x, SO_2, NO_2)	0.20	0.3
		$PM_{2.5}$=f(O_x, CO, SO_2, NO_2)	0.33	0.3
	"Northern" cities	$PM_{2.5}$=f(O_x)	0.11	0.7
		$PM_{2.5}$=f(O_x, SO_2)	0.29	0.8
		$PM_{2.5}$=f(O_x, NO_2)	0.20	0.5
		$PM_{2.5}$=f(O_x, CO)	0.54	0.7
		$PM_{2.5}$=f(O_x, SO_2, NO_2)	0.36	0.6
		$PM_{2.5}$=f(O_x, SO_2, NO_2, CO)	0.60	0.6
Deweathered	"2+26"cities	$PM_{2.5}$=f(O_x)	0.73	5.7
		$PM_{2.5}$=f(O_x, SO_2)	0.74	5.1
		$PM_{2.5}$=f(O_x, NO_2)	0.74	6.1
		$PM_{2.5}$=f(O_x, CO)	0.78	4.6
		$PM_{2.5}$=f(O_x, SO_2,NO_2)	0.76	5.4
		$PM_{2.5}$=f(O_x, SO_2, NO_2, CO)	0.82	2.8
	"Northern" cities	$PM_{2.5}$=f(O_x)	0.64	3.7
		$PM_{2.5}$=f(O_x, SO_2)	0.70	2.8
		$PM_{2.5}$=f(O_x, NO_2)	0.69	4.0
		$PM_{2.5}$=f(O_x, CO)	0.81	1.9
		$PM_{2.5}$=f(O_x SO_2, NO_2)	0.73	3.3
		$PM_{2.5}$=f(O_x, SO_2, NO_2, CO)	0.83	1.9

SI References

(1) Abadie, A.; Diamond, A.; Hainmueller, J. Comparative Politics and the Synthetic Control Method. *Am J Pol Sci* **2015**, *59* (2), 495–510. https://doi.org/10.1111/ajps.12116.

(2) Abadie, A.; Diamond, A.; Hainmueller, J. Synthetic Control Methods for Comparative Case Studies: Estimating the Effect of California's Tobacco Control Program. *J Am Stat Assoc* **2010**, *105* (490), 493–505. https://doi.org/10.1198/jasa.2009.ap08746.

(3) Abadie, A. Using Synthetic Controls: Feasibility, Data Requirements, and Methodological Aspects. *J Econ Lit* **2021**, *59* (2), 391–425. https://doi.org/10.1257/jel.20191450.

(4) Peng, Y.; Michael, B.; Aaron, C.; T, B. R.; Jiangmei, L.; Yunning, L.; Ruiming, L.; Weihua, W.; Jinlei, Q.; Lijun, W.; Maigeng, Z. Long-Term Fine Particulate Matter Exposure and Nonaccidental and Cause-Specific Mortality in a Large National Cohort of Chinese Men. *Environ Health Perspect* **2017**, *125* (11), 117002. https://doi.org/10.1289/EHP1673.

(5) Burnett, R.; Chen, H.; Szyszkowicz, M.; Fann, N.; Hubbell, B.; Pope, C. A.; Apte, J. S.; Brauer, M.; Cohen, A.; Weichenthal, S.; Coggins, J.; Di, Q.; Brunekreef, B.; Frostad, J.; Lim, S. S.; Kan, H.; Walker, K. D.; Thurston, G. D.; Hayes, R. B.; Lim, C. C.; Turner, M. C.; Jerrett, M.; Krewski, D.; Gapstur, S. M.; Diver, W. R.; Ostro, B.; Goldberg, D.; Crouse, D. L.; Martin, R. v; Peters, P.; Pinault, L.; Tjepkema, M.; van Donkelaar, A.; Villeneuve, P. J.; Miller, A. B.; Yin, P.; Zhou, M.; Wang, L.; Janssen, N. A. H.; Marra, M.; Atkinson, R. W.; Tsang, H.; Quoc Thach, T.; Cannon, J. B.; Allen, R. T.; Hart, J. E.; Laden, F.; Cesaroni, G.; Forastiere, F.; Weinmayr, G.; Jaensch, A.; Nagel, G.; Concin, H.; Spadaro, J. v. Global Estimates of Mortality Associated with Long-Term Exposure to Outdoor Fine Particulate Matter. *Proceedings of the National Academy of Sciences* **2018**, *115* (38), 9592. https://doi.org/10.1073/pnas.1803222115.

(6) Zhou, M.; Liu, H.; Peng, L.; Qin, Y.; Chen, D.; Zhang, L.; Mauzerall, D. L. Environmental Benefits and Household Costs of Clean Heating Options in Northern China. *Nat Sustain* **2021**. https://doi.org/10.1038/s41893-021-00837-w.

(7) Zhang, Q.; Zheng, Y.; Tong, D.; Shao, M.; Wang, S.; Zhang, Y.; Xu, X.; Wang, J.; He, H.; Liu, W.; Ding, Y.; Lei, Y.; Li, J.; Wang, Z.; Zhang, X.; Wang, Y.; Cheng, J.; Liu, Y.; Shi, Q.; Yan, L.; Geng, G.; Hong, C.; Li, M.; Liu, F.; Zheng, B.; Cao, J.; Ding, A.; Gao, J.; Fu, Q.; Huo, J.; Liu, B.; Liu, Z.; Yang, F.; He, K.; Hao, J. Drivers of Improved PM2.5 Air Quality in China from 2013 to 2017. *Proceedings of the National Academy of Sciences* **2019**, *116* (49), 24463. https://doi.org/10.1073/pnas.1907956116.

(8) T, B. R.; Arden, P. C.; Majid, E.; Casey, O.; S, L. S.; Sumi, M.; H, S. H.; Gitanjali, S.; Bryan, H.; Michael, B.; Ross, A. H.; R, S. K.; R, B. J.; G, B. N.; Haidong, K.; Francine, L.; Annette, P.-U.; C, T. M.; M, G. S.; Ryan, D. W.; Aaron, C. An Integrated Risk Function for Estimating the Global Burden of Disease Attributable to Ambient Fine Particulate Matter Exposure. *Environ Health Perspect* **2014**, *122* (4), 397–403. https://doi.org/10.1289/ehp.1307049.

(9) Global Burden of Disease Collaborative Network. *Global Burden of Disease Study 2019 (GBD 2019) Burden by Risk 1990-2019*; Seattle, United States of America, 2020. https://doi.org/10.6069/630D-5V32.

(10) Fan, M.; He, G.; Zhou, M. The Winter Choke: Coal-Fired Heating, Air Pollution, and Mortality in China. *J Health Econ* **2020**, *71*, 102316. https://doi.org/10.1016/j.jhealeco.2020.102316.
